# i-CRISPR: a personalized cancer therapy strategy through cutting cancer-specific mutations

**DOI:** 10.1186/s12943-022-01612-x

**Published:** 2022-08-16

**Authors:** Junfeng Jiang, Yuanyuan Chen, Li Zhang, Qishu Jin, Liujun Wang, Sha Xu, Kexin Chen, Li Li, Tao Zeng, Xingfei Fan, Tingting Liu, Jiaxi Li, Jinjiang Wang, Chaofeng Han, Fu Gao, Yanyong Yang, Yue Wang

**Affiliations:** 1 Histology and Embryology Department, Naval Medical University, 800, Xiangyin Road, 200433 Shanghai, People’s Republic of China; 2 Shanghai Key Laboratory of Cell Engineering, Naval Medical University, 800, Xiangyin Road, 200433 Shanghai, People’s Republic of China; 3 Department of Radiation Medicine, Faculty of Naval Medicine, Naval Medical University, 800, Xiangyin Road, 200433 Shanghai, People’s Republic of China; 4 Department of Pathology, Faculty of Medical Imaging Laboratory of Medical Imaging, Naval Medical University, 800, Xiangyin Road, 200433 Shanghai, People’s Republic of China; 5 Department of Histology and Embryology, Harbin Medical University, Harbin, 150086 China; 6 Department of Plastic Surgery, The First Affiliated Hospital of Naval Medical University, Shanghai, 200433 China; 7 The 901th Hospital of PLA Jiont Logistic Support Force, Hefei, 230031 China; 8 Department of Oncology, Tongren Hospital, Shanghai Jiao Tong University School of Medicine, Shanghai, 200336 China

**Keywords:** Cancer mutation sequencing, CRISPR-Cas9, Gene editing, DNA damage repair

## Abstract

Developing a strategy to specifically kill cancer cells without inducing obvious damage to normal cells may be of great clinical significance for cancer treatment. In the present study, we developed a new precise personalized strategy named "i-CRISPR" for cancer treatment through adding DNA damage repair inhibitors(i) and inducing cancer cell-specific DNA double strand breaks by CRISPR. Through in vitro and in vivo experiments, we confirmed the efficacy of this strategy in multiple cancer models and revealed the mechanism of cell death. Our strategy might provide a novel concept for precise cancer therapy.

**Supplementary Information:**

The online version contains supplementary material available at 10.1186/s12943-022-01612-x.

## Main text

Currently, cancer is mainly treated by surgery, chemotherapy and radiotherapy but still can not be completely cured in a large proportion of patients [1, 2]. Mostly, surgeries do not remove all cancer cells, and other strategies, such as radiotherapy and chemotherapy, often result in severe adverse effects on normal tissues when killing cancer cells [3, 4]. Therefore, developing a strategy to specifically kill cancer cells without inducing obvious damage to normal cells may be of great clinical significance for cancer treatment.

**Fig. 1:**
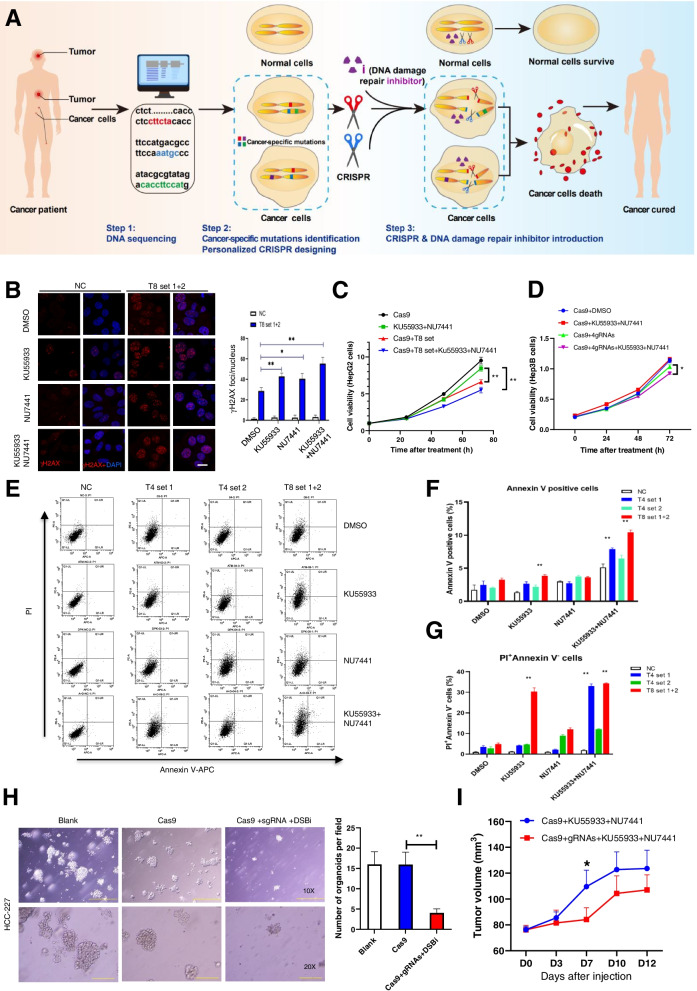
"i-CRISPR" strategy kills cancer cells by inducing DSBs in corresponding mutation sites. **A** Diagram of our proposed personalized CRISPR-mediated cancer treatment strategy named "i-CRISPR". The basic mechanism of radiotherapy is to induce DNA damage, especially DSBs, through radiation [5, 6]. When accumulated DNA breaks, especially DSBs, cannot be repaired, death signals are often activated. If DSBs are generated specifically in cancer cells through personalized CRISPR scissors and the repair of these DSBs is intensively blocked, specific killing of cancer cells may be achieved. Although the basic cell-killing mechanisms of our strategy and radiotherapy are similar, our strategy is more precise and personalized. Because both DNA damage repair inhibitors (**i**) and CRISPR are necessary, so we named this strategy "i-CRISPR". **B** Representative images of γH2AX foci in HepG2 cells at 48 h after transfection with three groups of gRNAs together with Cas9. To block the repair of DSBs, gene-edited cells were also treated with the ATM inhibitor KU55933 (10 μM), the DNA-PKcs inhibitor NU7441 (10 μM), or the combination of KU55933 (10 μM) + NU7441 (10 μM). Y: Quantitative analysis of the γH2AX foci number in the different groups indicated above. *P < 0.05. **P < 0.01. **C, D** At 0, 24, 48, and 72 h after gRNA transfection, HepG2 and Hep3B cells were pretreated with DMSO, KU55933, NU7441 and KU55933 + NU7441, and cell viability was determined with a CCK-8 assay at OD 450. **E** Representative images of cell apoptosis determined by flow cytometry analysis in cells transfected with three groups of gRNAs and Cas9 and treated with different inhibitors. **F, G** Quantitative analysis of cell apoptosis (Annexin V positive) and necrosis (PI positive, Annexin V negative) at 48 h after transfection combined with DNA repair inhibitor treatments. **H** Representative images of organoids (HCC-227) transfected with Cas9 and/or gRNAs combined with DNA damage repair inhibitor treatment. And the average number of organoids per field were quantified. **I** Tumor volume were recorded every three days after the injection of gRNA and DSB inhibitor. And tumor growth curve was obtained from the indicated two groups. *P < 0.05

The basic cell-killing mechanism of radiotherapy is to induce irreparable DNA damage, especially extensive DNA double strand breaks (DSBs), through ionizing radiation [5, 6]. The recent emergence of scissor-like CRISPR–Cas9 gene-editing technology makes it possible to precisely create DSBs at specific sites [7–9]. But in higher eukaryotes, DSBs are often repaired through nonhomologous end joining (NHEJ) and homologous recombination (HR) repair pathways [10, 11], and the role of CRISPR–Cas9-induced DSBs in cancer cells killing is largely unknown. Here, based on mutations in cancer cells identified with DNA sequencing, we propose a new personalized strategy using a customized CRISPR–Cas9 scissor system combined with DSB repair inhibitors (DSBRi) targeting NHEJ and HR (Fig. 1A), which we hypothesized would efficiently kill cancer cells with specific mutations without obviously affecting normal cells.

### An “i-CRISPR” strategy kills cancer cells by inducing DSBs in corresponding mutation sites

The designed workflow of our personalized CRISPR-mediated cancer treatment strategy is shown in Fig. 1A. In Step 1, tumor samples from patients are subjected to DNA sequencing to scan unique DNA mutations in patient cancer cells compared with normal cells. In Step 2, a group of customized guide RNAs (gRNAs) targeting these mutations but not recognizing any normal genome site are designed to develop an applicable CRISPR–Cas9 scissor systems. In Step 3, the scissor systems and DSBRi are introduced into cells, which leads to the specific killing of cancer cells.

To verify our strategy, we firstly analyzed the DNA sequences of the human hepatocellular carcinoma (HCC) cell line HepG2 through whole-genome sequencing and identified 3,985,698 single nucleotide variants (SNV) and 817,723 insertion-deletion mutations (indels) (Fig. S1). It was estimated that more than 1000 sites might have the potential to become CRISPR targets suitable for our strategy. In current study, we select 8 candidate target sites to design corresponding gRNA-Cas9 expressing adenoviruses, which were added into HepG2 cells in the following combinations: negative control (NC), 4 targets set 1 (T4-set1), 4 targets set 2 (T4-set2) and 8 targets set (T8-set). The cutting sites were further verified with Sanger sequencing in HepG2 cells transfected with the T8-sets (Fig. S2). As a classic DSB marker, phosphorylation of histone 2AX (γH2AX) was detected with immunofluorescence staining to monitor the induced DSBs. As expected, more γH2AX was significantly induced in all the gRNA-Cas9-expressing adenovirus-infected groups compared to the NC group respectively (Fig. S3A, B), with the most foci number in the T8-set (Fig. S3A, B). Most notably, we also added these gRNA-Cas9-expressing adenoviruses to Huh-7 cells without these mutants, and few DSB foci were observed (Fig. S3A).

To promote DSBs-induced cell death, we then applied chemical inhibitors targeting NHEJ with the DNA-PKcs inhibitor NU7441 and targeting HR repair with the ATM inhibitor KU55933 [12, 13], and DSBs repair were significantly suppressed by these inhibitors (Fig. 1B). Moreover, compared to others groups, most unrepaired DSBs were observed in ATM and DNA-PKcs both inhibited cells using NU7441 + Ku55933 (2i) (Fig. 1B). For cell survival analysis, we used two HCC cell lines HepG2 and Hep3B. Our data showed that the three gRNA groups inhibited cell viability in HepG2 (Fig. 1C, S3C-F). Moreover, when DNA repair was blocked by these DSBRi sets, cell viability was significantly suppressed (Fig. 1C, S3C-F), especially in cells treated with T8-set gRNAs-Cas9 in combination with the 2i (Fig. 1C). The similar strategy was also tested in Hep3B cells with another set of specific gRNAs, and the cell survival was also significantly suppressed (Fig. 1D). Alternatively, flow cytometry was further used to detect cell apoptosis in gene-edited HepG2 cells. Transfection of either T4-set or T8-set combined with one or two DNA repairing inhibitors significantly increased the percentage of Annexin V-positive cells as well as the number of PI(+)Annexin V (−) cells comparing to the control group. And most cell death was observed in the T8-set group combined with 2i again (Fig. 1E, F, G). Immunofluorescence also showed that the T8-set increased the cleavage of Caspase 3 (c-Caspase 3), which was further increased when 2i was applied (Fig. S4A-C), suggesting a possible role of caspase dependent apoptosis.

For more extensive validations of our strategy in other types of cancer cells or different forms of administration, we additionally employed another CRISPR scissor cell-killing model using a human prostate cancer (PCa) cell line DU145 treated with gRNA expressing lentiviruses and wortmannin, another DNA damage repair inhibitor both for NHEJ and HR (Fig. S5, 6). After joint analysis with public database, we selected 7 sites to design the corresponding gRNA-Cas9 lentivirus systems, which were verified with Sanger sequencing (Fig. S6A, B & Supplementary Table 1). A CCK-8 assay was also performed and the results showed that the simultaneous addition of Cas9 with gRNAs and wortmannin significantly inhibited cell viability (Fig. S6C). These combined treatments also significantly increased DU145 cell apoptosis (Fig. S7A, B). Moreover, more apoptotic cells were observed in wortmannin group compared to the 2i group, indicating that DNA damage repair was more extensively inhibited in wortmannin treated cells (Fig. S7A, B). However, further investigations with this group gRNAs did not show any inhibitory effect on viability on 293 T cells, a normal cell line without designed cutting sites (Fig. S7A-C). To investigate whether cancer cells develop resistance to this therapeutic strategy, DU145 cells were treated with a first round of 4gRNA treatment, and the survived cells further treated with the same 4gRNA or a different 3gRNAs for the second round. Our data showed that a second round of administration with different gRNAs induced more cell apoptosis (Fig. S8A, B) and further inhibited cell survival (Fig. S8C) compared to the 4gRNA single administration group.

Moreover, in vivo experiments in tumor-bearing nude mice also indicated that the “i-CRISPR” strategy inhibited the tumor growth of DU145 cells (Fig. S6C). In patient-based preclinical models, we established organoids model and PDX model to investigate the potential efficacy of our strategy. An established HCC organoid (HCC-227) with DNA sequencing data was cultured and their corresponding gRNAs were designed. Our data showed that CRISPR cutting of 3 sites combined with 2i treatment significantly inhibited the survival and average number of organoids (Fig. 1H). However, the gRNAs targeting mutation sites in HCC-227 showed no influence on another organoid, HCC-12 (Fig. S9A, B). In PDX model, the tumors were injected intratumorally with Cas9&gRNAs lentivirus plus 2i, with Cas9-lentivirus plus 2i as the control group. We found that tumor growth in the treatment group was significantly inhibited, compared with the control group (Fig. 1I). In addition, no significant difference was observed in body weight (Fig. S10A), as well as parameters in blood routine test or biochemical analysis (Fig. S10B-I). These results suggested that our treatment strategy was unlikely to cause serious safety problems on mouse models. Consistent with this preliminary conclusion, we did not find a significant increased off-targets in DU145 processed by our strategy through GUIDE-seq technology (Fig. S11) [14].

All these in vivo and in vitro results indicated that site-specific gene editing of mutations with DNA repair inhibitors dramatically inhibited tumor growth and it might have application value in tumor treatment in the future. In addition, we also verified the cutting efficacy with Sanger sequencing when all the gRNAs and Cas9 were delivered, and sometimes one or two sites could not be edited efficiently, which may be due to the introduction efficacy or site selection. Thus, cutting sites selection and the cutting efficacy of each single mutation sites were required to be optimized in future study.

### The molecular mechanism of the “i-CRISPR” strategy on cell killing

**Fig. 2:**
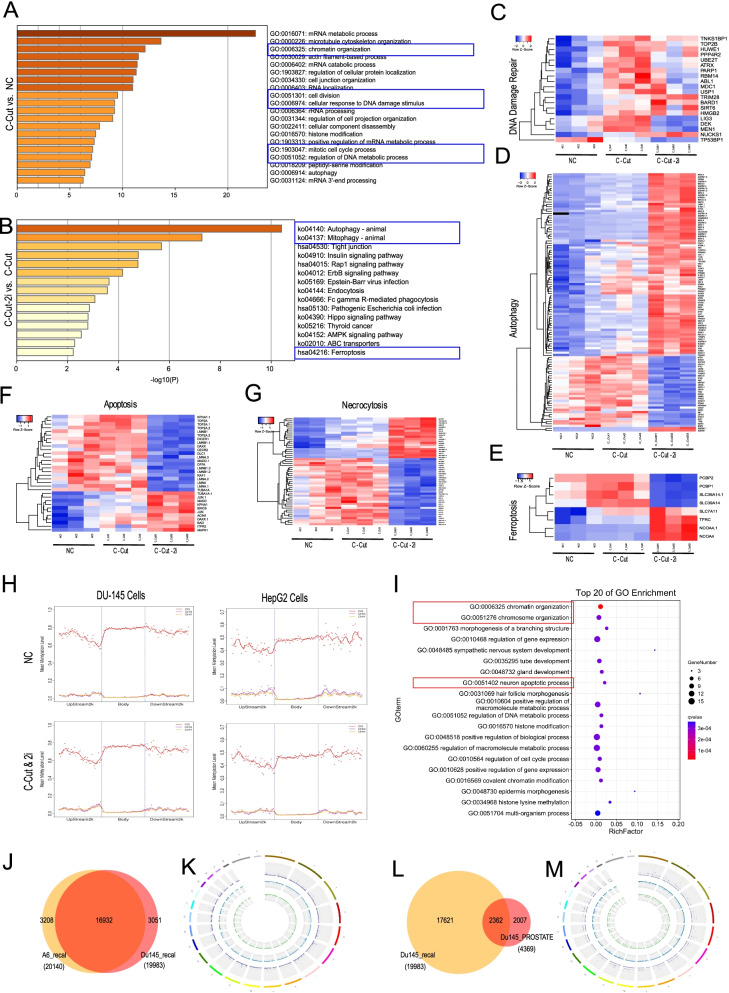
The molecular mechanism of the "i-CRISPR" strategy on cell killing. **A, B** Enrichment of GO terms for the proteins with upregulated phosphorylation between the C-Cut and NC groups (A), C-Cut-2i and C-Cut groups (**B**). Differentially phosphorylated proteins were tested by quantitative phosphoproteomics analysis using tandem mass spectrometry. NC: negative control, only treated with Cas9; C-Cut: CRISPR-Cut, treated with Cas9 and the 8 gRNAs in the T8-set; C-Cut-2i，CRISPR-Cut treated with the T8-set and 2i. **C-G** Heatmap shows the selected differentially phosphorylated proteins related to DNA damage repair (**C**), autophagy (**D**), ferroptosis (**E**), apoptosis (**F**), and necrocytosis (**G**) among the 3 groups. **H** Analyses of whole-genome DNA methylation patterns in body regions (body), upstream-2 k (− 2 k) regions and dowstream-2 k (2 k) regions of genes in control and treated HepG2 cells and DU145 cells. NC: negative control, only treated with Cas9; C-Cut&2i，CRISPR-Cut with 8gRNAs (for HepG2) or 7gRNAs(for DU145) and 2i (NU7441 and KU55933). **I** Enrichment of GO terms for the genes that have differentially methylated regions (DMRs) on C in both DU145 and HepG2(refer to Fig. S8C). **J**. Venn diagram shows the common and specific mutations in DU145 and A6 discovered by whole genome sequencing (WGS). A6 is a single-cell clone derived from DU145 that has been cultured alone for approximately 60 passages in our laboratory. **K** Circos plot showing the distribution of the common and specific mutations in DU145 and A6. Purple: common mutations in DU145 and A6; blue: specific mutations found only in A6; green: specific mutations found only in DU145. **L** Venn diagram shows the common and specific mutations in DU145 and DU145 public data. Public DU145 data are public DU145 mutation data (CCLE, https://depmap.org/portal/cell_line/ACH-000979?tab=mutation). **M** Circos plot showing the distribution of the common and specific mutations in DU145 and DU145 public data. Purple: common mutations in DU145 and DU145 public data; Blue: specific mutations found only in DU145; Green: specific mutations found only in DU145 public data

To explore the cancer cell killing mechanism of our strategy, we conducted quantitative phosphoproteomics using tandem mass spectrometry on three groups of HepG2 cells: (1) NC, negative control; (2) C-Cut, CRISPR-Cut with T8-set; and (3) C-Cut-2i, CRISPR-Cut with T8-set and 2i. We found that, although there were only few differentially phosphorylated proteins among C-Cut group and the NC group (Fig. S12A & S13A-C), C-Cut-2i group had an unique phosphorylated protein pattern much differentially from the other two groups (Fig. S12B-C & S13A-C). All the differentially phosphorylated proteins were located primarily in the nucleus (Fig. S13D). In particular, compared to the NC group, most of the altered proteins in the C-Cut group showed increased phosphorylation rather than decreased phosphorylation (Fig. S12A), and they were enriched in chromatin organization and DNA damage-related pathways after GO analysis (Fig. 2A&C). Nearly all the altered DNA damage-related proteins in the C-Cut group showed increased phosphorylation levels but could be suppressed by adding the two inhibitors (Fig. 2C). Interestingly, differing significantly from those in the other two groups, the differentially phosphorylated proteins in the C-Cut-2i group were greatly enriched in various cell death-related pathways, including autophagy, ferroptosis, apoptosis, necrocytosis, and necroptosis, especially autophagy (Fig. 2B&D-G & Fig. S12D-E, S14-16). We therefore suggest that the activation of autophagy may play critical role in the molecular mechanism for the increased cell death in the C-CUT-2i group, as described above.

In addition, we also examined the effect of our strategy on DNA methylation related epigenetic regulations in DU145 and HepG2 cells by whole genome bisulfite sequencing (WGBS). We found that our strategy by gRNA-Cas9 and DSBRis did not obviously alter the distribution of DNA methylation regions in both the two cell lines (Fig. 2H). However, there were some differentially methylated regions (DMRs) in DU145 cells treated by our strategy comparing to the control cells, especially for the methylation on CHH and C (Fig. S17A-B). Interestingly, these DMRs located genes were also enriched in cell death, programed cell death and chromosome organization pathways after GO analysis (Fig. S17C-D). Similar results were also presented in HepG2 (Fig. S18). The common DMRs located genes both in DU145 and HepG2 were also enriched in chromosome organization and apoptotic process (Fig. 2I& S19). As two examples of DMRs located genes possibly contributing to cell viability, we found that the methylation of the JAK2 gene was significantly increased, and the methylation of the FAS gene was significantly decreased after being treated with our strategy (Fig. S20).

Another difficult problem faced by cancer therapy is the evolution of cancer cells with continuous mutations. In the regard, the therapeutic efficiency of our strategy should be determined by the existence of invariable mutations among all the generations of cancer cells. To verify this, we performed whole-genome sequencing on a DU145 single-cell clone A6, which was cultured alone for approximately 60 passages in our laboratory. There were 3208 new mutations in this single cell clone, but 16,932 (81.05%) mutations remained unchanged compared with ordinary DU145 (Fig. 2J-K). Next, we performed WGS on additional two DU145 single-cell clone B12 and B13, which were cultured alone for approximately 80 passages in our laboratory. By comparing the three clones, it can be seen that these single-cell clones are continuously evolving, generating new mutations and showing heterogeneity (Fig. S21A-B). However, among all the mutations in each clone, only a small part is unique to the respective clone (A6: 17.11%; B12: 32.10%, B13: 38.72%), and most of the original mutations are retained (Fig. S21A-B). Comparing the three clones together with DU145, it can still be found that most of the mutations were shared, and all the 7gRNAs targeted mutations could be detected in all the 4 sample (Fig. S21B-C).

Moreover, we also compared our DU145 data with public DU145 mutation data (CCLE, https://depmap.org/portal/cell_line/ACH-000979?tab=mutation) released many years ago and found that more than half of the mutations were still retained (Fig. 2L-M). These results suggested that if we choose 10 mutation sites as the targets for our strategy, the possibility that all 10 sites would become ineffective in the process of cancer evolution would be extremely low.

In addition, we also analyzed whether “i-CRISPR” has practical feasibility in patients. As we previously reported, the mutation burden was 1.0 per megabase (Mb), and the median substitution rate was 1.4 per Mb in our cohort of 208 prostate cancer patients [15]. There were more than 100 mutation sites suitable to be targets of our strategy in each prostate cancer patient through rough estimation. Analysis of the data of 2554 European prostate cancer patients [15] also suggested that on average, there are more than 100 DNA mutation sites suitable for CRISPR-specific cleavage in each patient.

These results also suggest that our strategy has great advantages in solving the cancer evolution problem faced by current cancer therapy treatments. Moreover, with the future development of sequencing and bioinformatics technologies such as clonal evolutionary analysis, it will be possible to identify the original mutations which is universal in all the cancer cells from one patient. Using our strategy to specifically targeting these original mutations may also overcome the problems caused by cancer heterogeneity.

## Conclusion

Our study presented a precise cancer treatment strategy through inducing cancer cell-specific DSBs and subsequent cell death by a CRISPR system combined with DNA damage repair inhibitors, which provides a novel concept for personalized cancer therapy.

## Supplementary Information

**Additional file 1: Figure S1.** WGS results in HepG2. **Figure S2.** Representative Sanger sequencing results of gRNA-expression vectors and their targeting sites in HepG2 and Hep3B. **Figure S3.** The "i-CRISPR" strategy could induce DSBs and cell death in HepG2 cells. **Figure S4.** Targeted induction of DSB resulted in apoptosis in cancer cells carrying specific mutations. **Figure S5.** WGS results in DU145. **Figure S6.** The "i-CRISPR" strategy could suppress DU145 cell viability. **Figure S7.** The "i-CRISPR" strategy could induce cell death in DU145 cancer cells but not normal 293 T cells. **Figure S8.** CRISPR targeting new targets could overcome the possible drug resistance problems of our strategy. **Figure S9.** The "i-CRISPR" strategy could only specifically suppress HCC organoid with the designed mutations. **Figure S10.** The "i-CRISPR" strategy could not significantly affect the weight, blood routine and other biochemical indicators of PCa PDX mice. **Figure S11.** Sequences of off-target sites identified by GUIDE-seq for the mixture of Cas9 and 3 gRNAs targeting mutated sites and treated DU145 cells. **Figure S12.** Quantitative phos-phoproteomics analysis elucidate the molecular mechanism of the "i-CRISPR" strategy in HepG2 cells. **Figure S13.** Differentially phosphorylated proteins analysis after the "i-CRISPR" strategy treatment. **Figure S14.** Enrichment of GO terms for differentially phosphorylated proteins among the 3 groups. **Figure S15.** Activation of autophagy may play critical role in the molecular mechanism for the increased cell death of our strategy-part 1. **Figure S16.** Activation of autophagy may play critical role in the molecular mechanism for the increased cell death of our strategy-part 2. **Figure S17.** DNA methylation analysis in control and treated DU145 cells. **Figure S18.** DNA methylation analysis in control and treated HepG2 cells. **Figure S19.** Joint analysis of the DNA methylation data in DU145 and HepG2 cells. **Figure S20.** Gene body region methylation of representative DMR located 312 genes in DU145 and HepG2 cells. **Figure S21.** Whole-genome sequencing results of DU145 and the 3 DU145 derived single cells (A6, B12, B13) cultured in our laboratory. Supplementary materials and methods.

**Additional file 2.**

## Data Availability

The datasets used and analyzed during the current study are available within the manuscript and its additional files.

## Abbreviations

CRISPR: Clustered Regularly Interspaced Short Palindromic Repeats
DSB: DNA double strand breaks
DSBRi: DSB repair inhibitors
NHEJ: Non-homologous end joining
HR: Homologous recombination
ATM: Ataxia telangiectasia mutated
DNA-PK: DNA-dependent protein kinase
PI3KK: PI3K-like kinase
HCC: Hepatocellular carcinoma
PCa: Human prostate cancer
GO: Gene Ontology
SNV: Single nucleotide variants
Mb: Megabase
WGBS: Whole genome bisulfite sequencing
DMRs: Differentially methylated regions
