# Supplementary material for: i-CRISPR: a personalized cancer therapy strategy through cutting cancer-specific mutations
Source: Mol Cancer. 2022 Aug 16;21:164. doi: 10.1186/s12943-022-01612-x (PMC9380384; doi:10.1186/s12943-022-01612-x)
Supplement: Supplementary file 1 — Additional file 1: Figure S1. WGS results in HepG2. Figure S2. Representative Sanger sequencing results of gRNA-expression vectors and their targeting sites in HepG2 and Hep3B. Figure S3. The "i-CRISPR" strategy could induce DSBs and cell death in HepG2 cells. Figure S4. Targeted induction of DSB resulted in apoptosis in cancer cells carrying specific mutations. Figure S5. WGS results in DU145. Figure S6. The "i-CRISPR" strategy could suppress DU145 cell viability. Figure S7. The "i-CRISPR" strategy could induce cell death in DU145 cancer cells but not normal 293 T cells. Figure S8. CRISPR targeting new targets could overcome the possible drug resistance problems of our strategy. Figure S9. The "i-CRISPR" strategy could only specifically suppress HCC organoid with the designed mutations. Figure S10. The "i-CRISPR" strategy could not significantly affect the weight, blood routine and other biochemical indicators of PCa PDX mice. Figure S11. Sequences of off-target sites identified by GUIDE-seq for the mixture of Cas9 and 3 gRNAs targeting mutated sites and treated DU145 cells. Figure S12. Quantitative phos-phoproteomics analysis elucidate the molecular mechanism of the "i-CRISPR" strategy in HepG2 cells. Figure S13. Differentially phosphorylated proteins analysis after the "i-CRISPR" strategy treatment. Figure S14. Enrichment of GO terms for differentially phosphorylated proteins among the 3 groups. Figure S15. Activation of autophagy may play critical role in the molecular mechanism for the increased cell death of our strategy-part 1. Figure S16. Activation of autophagy may play critical role in the molecular mechanism for the increased cell death of our strategy-part 2. Figure S17. DNA methylation analysis in control and treated DU145 cells. Figure S18. DNA methylation analysis in control and treated HepG2 cells. Figure S19. Joint analysis of the DNA methylation data in DU145 and HepG2 cells. Figure S20. Gene body region methylation of representative DMR located 31 [file 12943_2022_1612_MOESM1_ESM.pdf]

Supplementary Information

i-CRISPR：a personalized cancer therapy strategy through cutting cancer-specific mutations

Junfeng Jiang[#1,4], Yuanyuan Chen[#2], Li Zhang[#3], Qishu Jin[5], Liujun Wang[1], Sha Xu[1], Kexin Chen[6], Li Li[1], Tao Zeng[1,7], Xingfei Fan[1], Tingting Liu[2], Jiaxi Li[8], Jinjiang Wang[8], Chaofeng Han*[1], Fu Gao*[2], Yanyong Yang*[2], Yue Wang*[1,4]

[#]*Authors contributed equally to this work.*

1. Histology and Embryology Department, Naval Medical University, Shanghai, 200433, China
2. Department of Radiation Medicine, Faculty of Naval Medicine, Naval Medical University, 800, Xiangyin Road, 200433, Shanghai, P.R. China;
3. Department of Pathology, Faculty of Medical Imaging Laboratory of Medical Imaging, Naval Medical University, Shanghai, 200433, China
4. Shanghai Key Laboratory of Cell Engineering, Naval Medical University, Shanghai, 200433, China.
5. Department of Histology and Embryology, Harbin Medical University, Harbin 150086, China
6. Departmentof Plastic Surgery, The First Affiliated Hospitalof Naval Medical University, Shanghai 200433, China
7. The 901th Hospital of PLA Jiont Logistic Support Force, HeFei, 230031, China
8. Department of Oncology, Tongren Hospital, Shanghai Jiao Tong University School of Medicine, Shanghai, 200336, China

*Corresponding author:

Yue Wang, wangyuesmmu@163.com; Yanyong Yang, yyyang2010@163.com;

Fu Gao, gaofusmmu@163.com; Chaofeng Han, hcf@immunol.org;

Address: 800, Xiangyin Road, 200433, Shanghai, P.R. China.

1. Supplementary figures

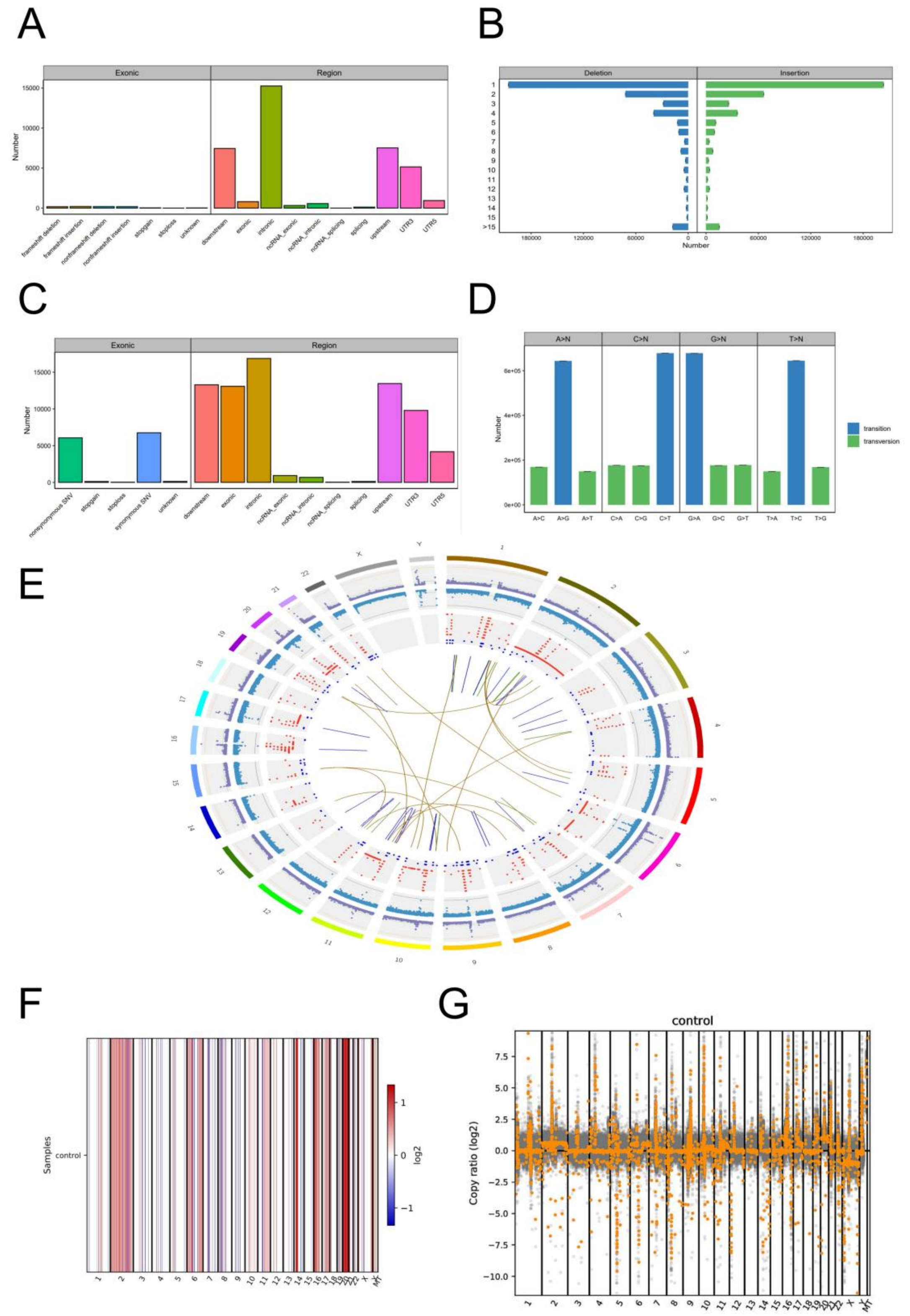

Fig. S1 WGS results in HepG2.

A. The WGS (whole genome sequencing) results showed the distribution of indel (insertion or deletion mutations) on different DNA segments in HepG2 cells.

B. The indel substitution in HepG2 cells discovered by WGS.

C. The WGS results showed the distribution of SNV on different DNA segments in HepG2 cells.
D. The SNV substitution in HepG2 cells discovered by WGS.
E. A Circos plot showing the genomic landscape of mutations (including SNVs, Indels, CNVs, and gene fusions) in HepG2 cells discovered by WGS. The outer ring shows chromosome ideograms. The bars along each inner ring represent mutation events.
F-G. Copy-number variation (CNV) distribution on chromosomes in our cultured HepG2 cells discovered by WGS.

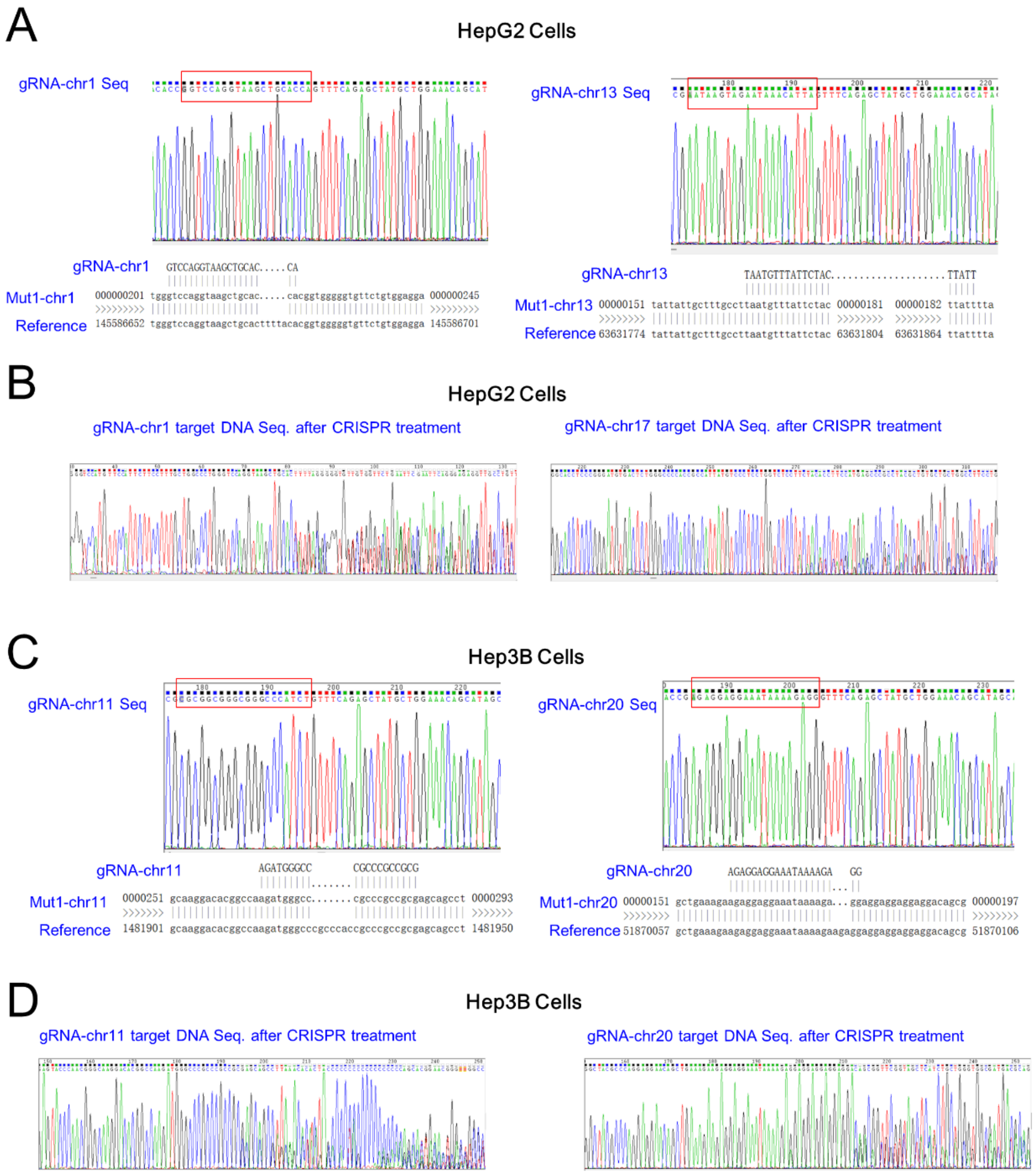

Fig. S2 Representative Sanger sequencing results of gRNA-expression vectors and their targeting sites in HepG2 and Hep3B.

A. The representative sequences of the designed gRNA-expression vectors which only target mutated DNA sites in hepatocellular carcinoma (HCC) cell line HepG2.

B. Representative Sanger sequencing results of the PCR product showed the emergence of abnormal peaks at the gRNA targeting site, which indicated the designed CRISPR could successful induce DSBs in HePG2 cells. This experiment was used to verify the CRISPR cutting efficiency. The cells only received CRISPR treatment, but not DSB repair inhibitors (DSBRi) treatment.

C. The representative sequences of the designed gRNAs which only target mutated DNA sites in HCC cell line Hep3B.

D. Representative Sanger sequencing results of the PCR product showed the emergence of abnormal peaks at the gRNA targeting site, which indicated the designed CRISPR could successful induce DSBs in HeP3B cells.

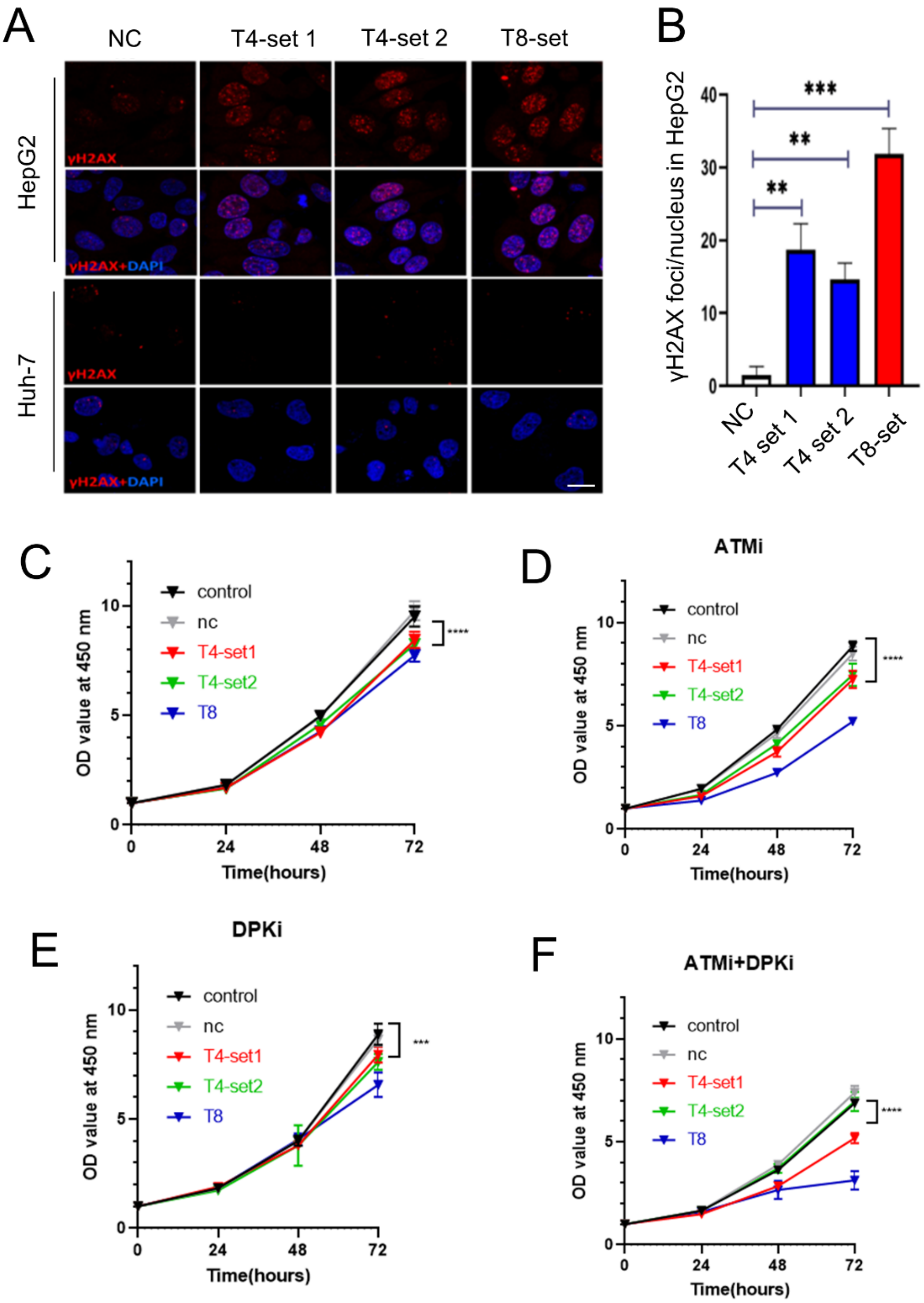

Figure S3 The "i-CRISPR " strategy could induce DSBs and cell death in HepG2 cells.

A. Representative images of γH2AX foci in HepG2 cells at 48 h after transfection with three groups of gRNAs together with Cas9. Scale bar, 20μm.

B. Quantitative analysis of the γH2AX foci number in the different groups indicated above. *P<0.05. **P<0.01.

C-F. At 0, 24, 48, and 72 h after gRNA transfection, cells were pretreated with DMSO (C), KU55933 (D), NU7441 (E) and KU55933+NU7441 (F), and cell viability was determined with a CCK-8 assay at OD450.

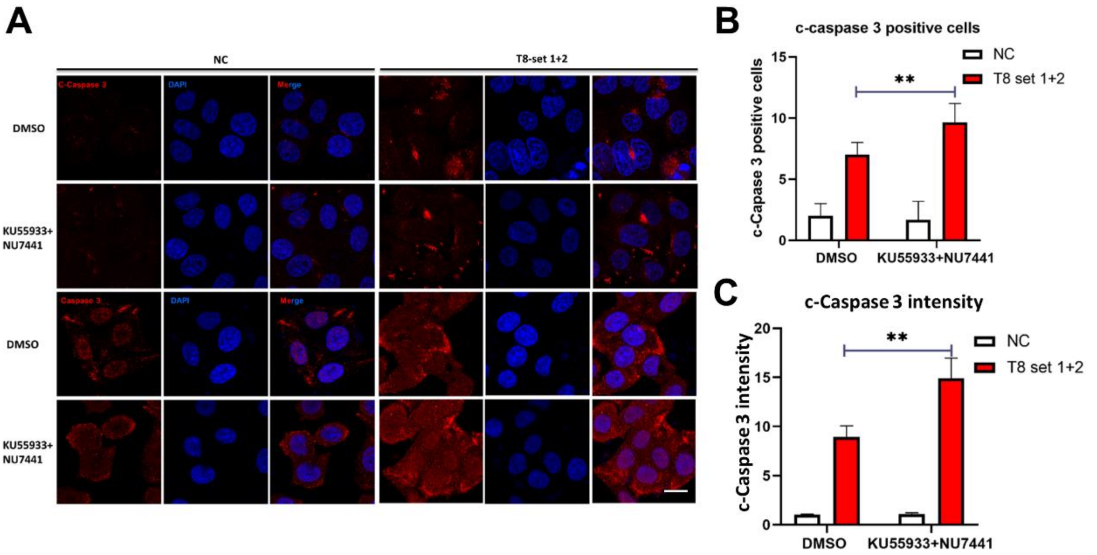

Figure S4. Targeted induction of DSB resulted in apoptosis in cancer cells carrying specific mutations.

A-C. representative images and quantitative analysis of cleaved Caspase 3 (c-Caspase 3) in T8 gRNAs&Cas9 introduced group with/without DNA damage repair treatments (KU55933+NU7441). Scale bar, 20μm. *P<0.05. **P<0.01.

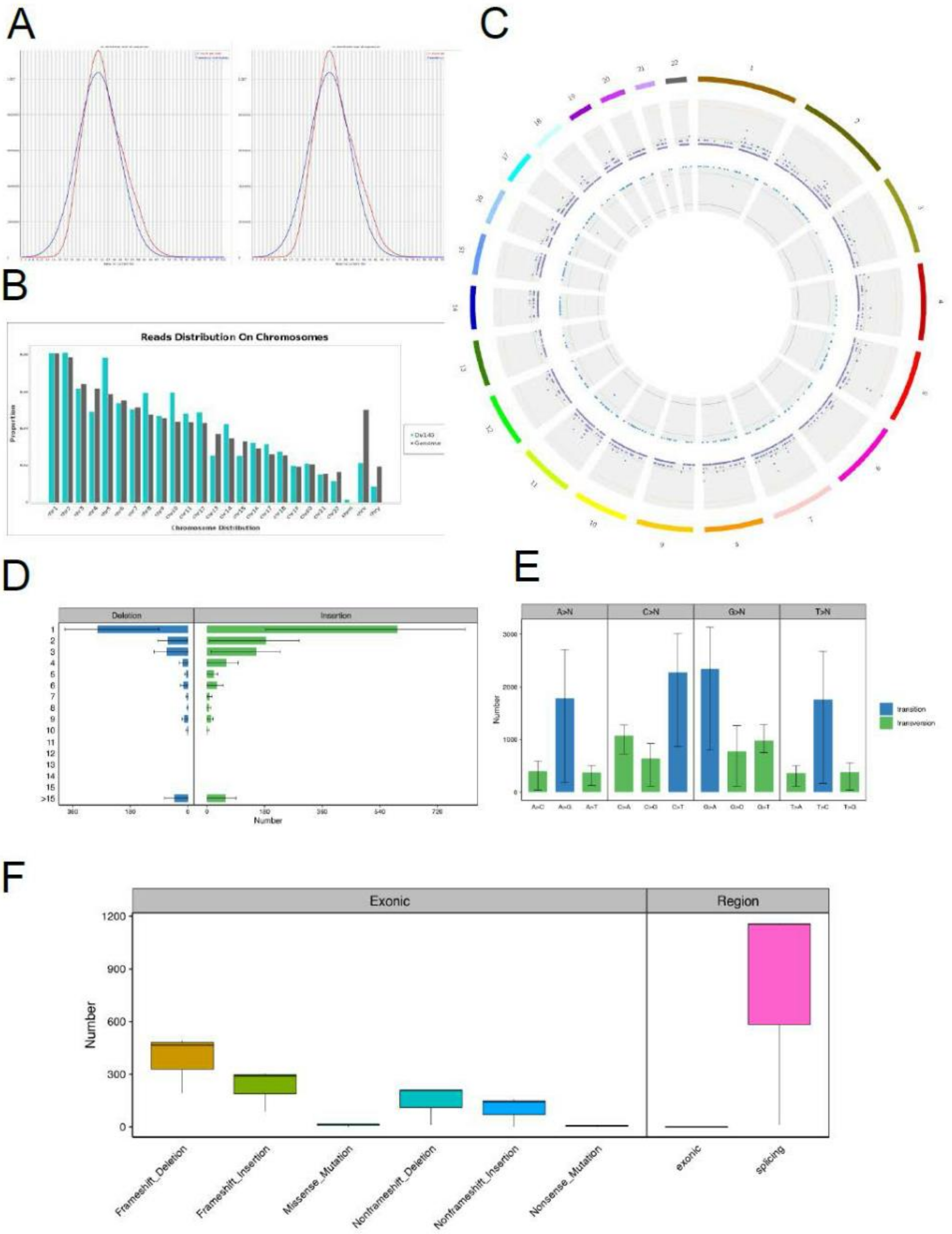

Figure S5. WGS results in DU145.

A. Sequence GC Content in DU145 discovered by WGS (whole genome sequencing).

B. Reads distribution on chromosomes in DU145 discovered by WGS.

C. A Circos plot showing the genomic landscape of in our cultured DU145 cells discovered by WGS. The outer ring shows chromosome ideograms. The bars along each inner ring represent mutation events.

D. WGS results showed the substitution of indel (insertion or deletion mutations) in our cultured DU145 cells.

E. SNP substitution in our cultured DU145 cells discovered by WGS.

F. WGS results showed the distribution of indel on different DNA segments in our cultured DU145 cells.

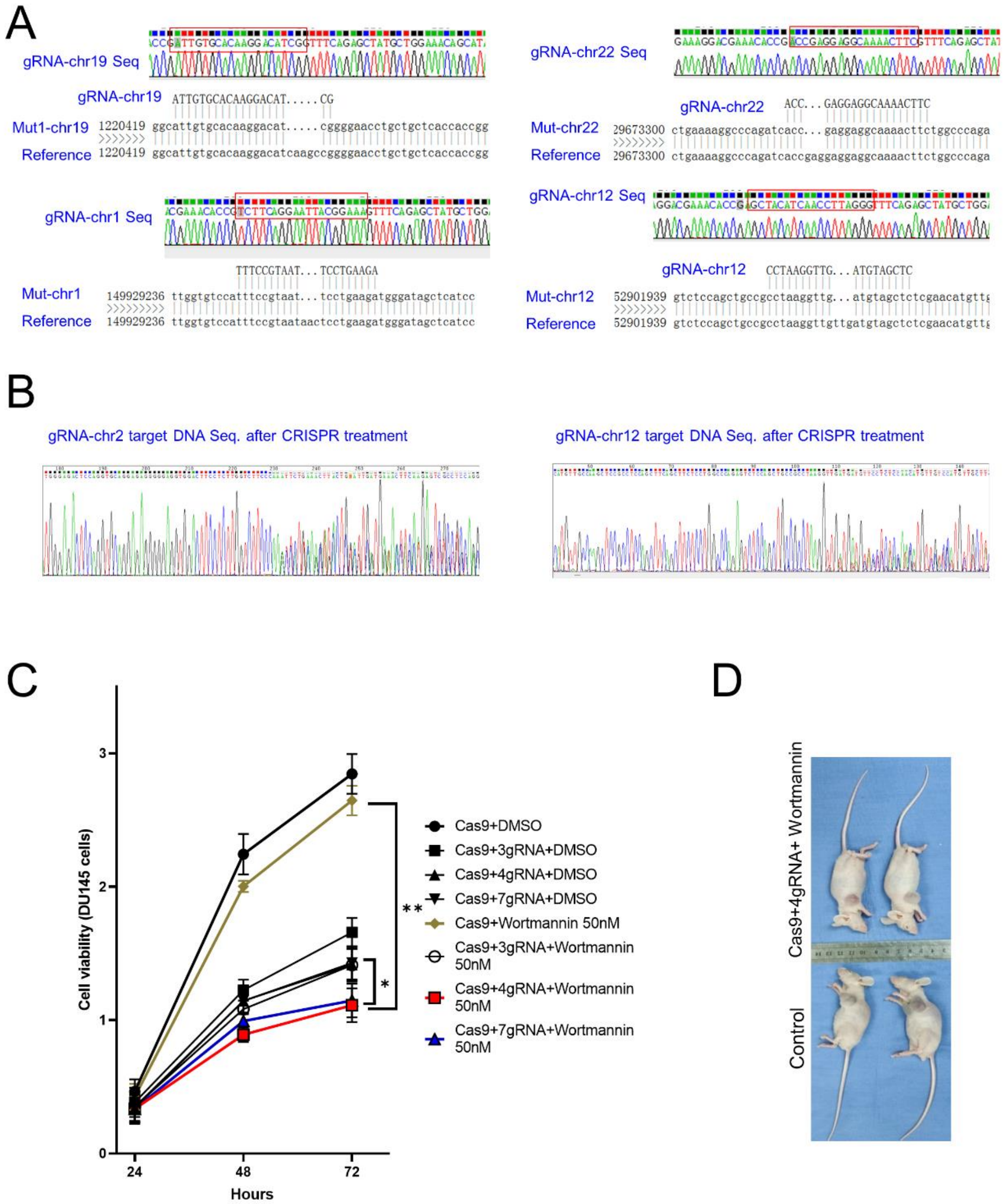

Figure S6. The "i-CRISPR " strategy could suppress DU145 cell viability.

A. The representative sequences of the designed gRNAs which only target mutated DNA sites in Prostate cancer cells DU145.

B. Representative Sanger sequencing results of the PCR product of Cas9 and gRNA introduced DU145 cells showed the emergence of abnormal peaks at the gRNA targeting sites, which indicated the designed CRISPR could successful induce DSBs in cells.

C. CCK-8 assay results of cell viability of DU145 cells after the treatment of Cas9 and different combinations of gRNA-expressing lentiviruses and Wortmannin (50nM).

D. Xenograft experiments showed decreased growth of DU145 cells after 4gRNAs-Cas9 and Wortmannin treatment in nude mice.

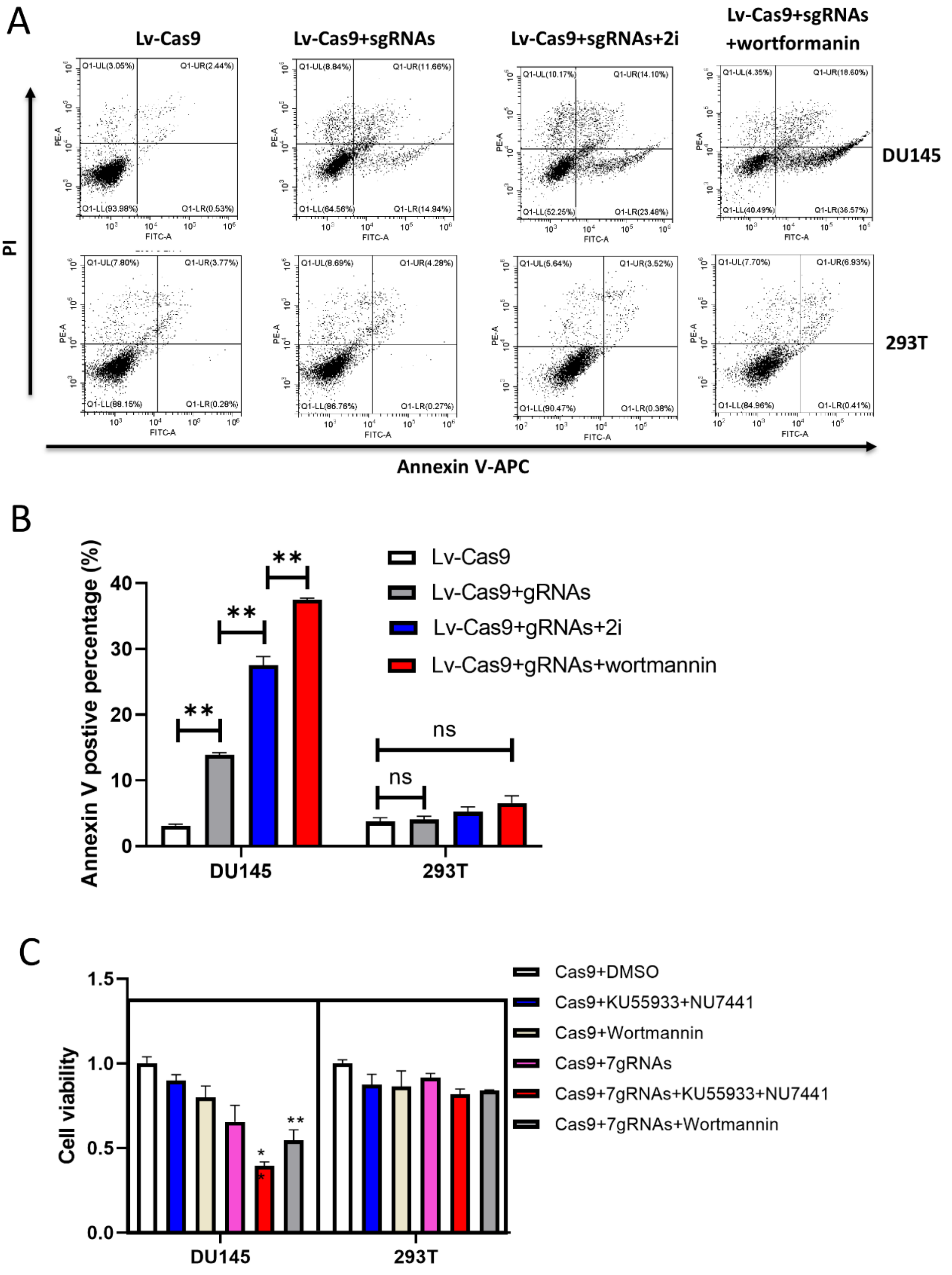

Figure S7. The "i-CRISPR" strategy could induce cell death in DU145 cancer cells but not normal 293T cells.

A. Representative of cell apoptosis in DU145 cells transfected with a gRNAs combined treatment with DNA damage repair inhibitors. A lv-cas9 was used as a negative control. And NU7441+KU55933 and wortmannin was used as DSB inhibitors. A normal 293T cells without the relative cutting sites were used to determine the specificity of this strategy.

B. Quantification of apoptotic cells from different groups were shown. **P<0.01 Vs the relative group.

C. DU145 cells and 293T cells transfected with lv-cas9, or lv-cas9 plus sgRNAs, and treated with DNA damage repair inhibitors. After 72h later, cell viability was measured in DU145 cells and 293T cell with a CCK-8 assay.

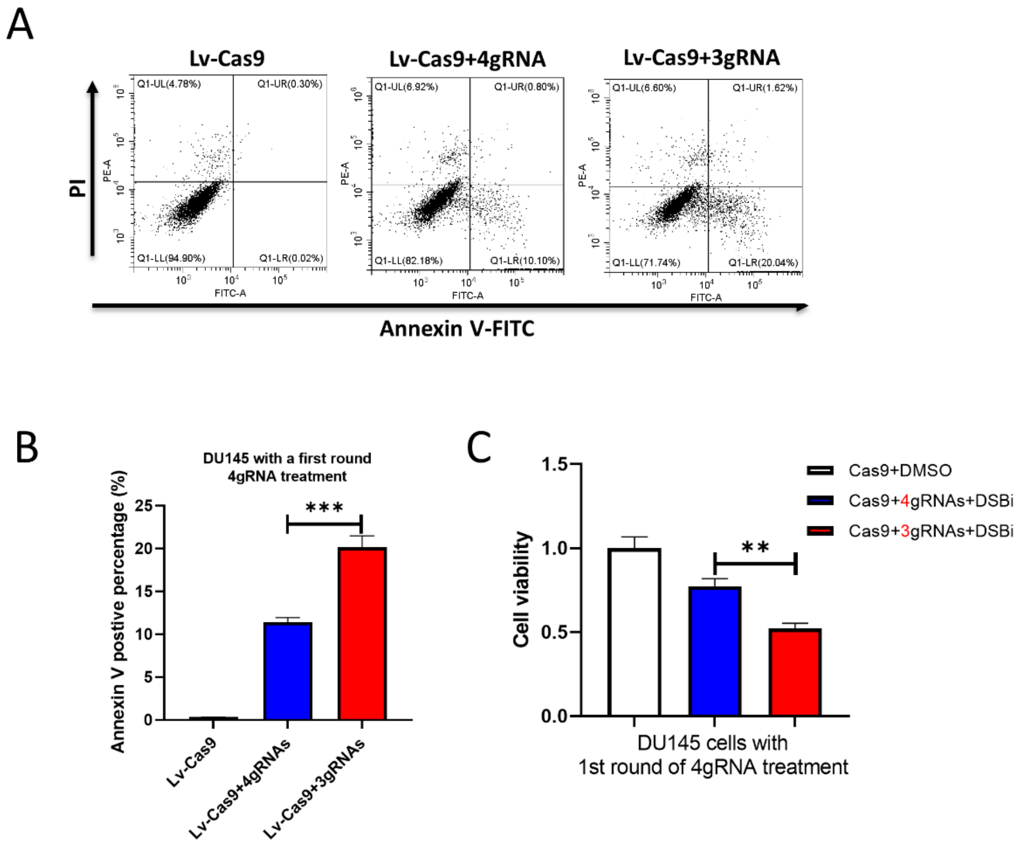

Figure S8 CRISPR targeting new targets could overcome the possible drug resistance problems of our strategy.

A. To test the possible resistance of the strategy, DU145 were treated with a first round of 4 gRNAs mediated CRISPR cutting. Then the survived cells were subjected to the same 4 gRNAs or a different group of 3 gRNAs cutting. Cell apoptosis were measured with flowcytometry assay. Representative of cell apoptosis in DU145 cells transfected with two round of gRNAs was shown in Fig. S7A.

B. Quantification of apoptotic cells from different groups were shown. ***P<0.001 Vs the 4gRNAs group.

C. Cell viability was measured with CCK-8 assay in DU145 cell treated with two rounds of CRISPR cutting treatments. **P<0.01 Vs the 4gRNAs group.

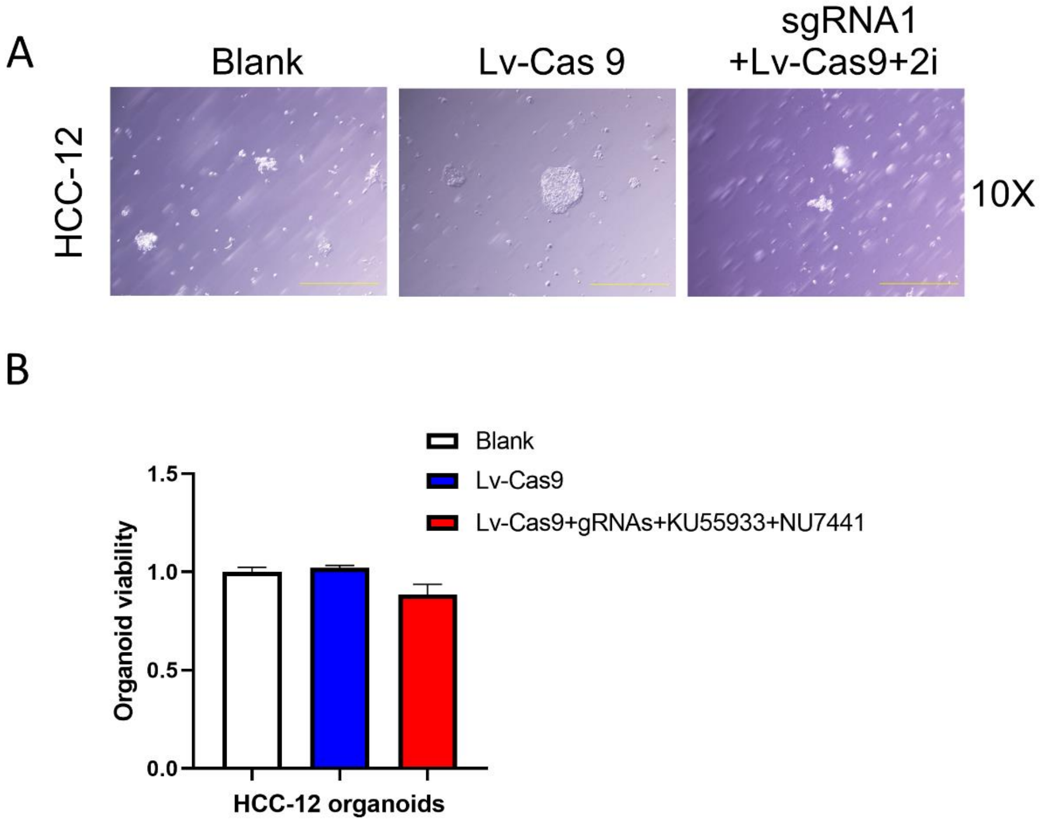

Figure S9. The "i-CRISPR " strategy could only specifically suppress HCC organoid with the designed mutations.

A. Representative images of organoids (HCC-12) transfected with cas9 and/or gRNAs (HCC-227) combined with DNA damage repair inhibitor treatment.

B. The viability of organoids was measured with a CCK-8 assay.

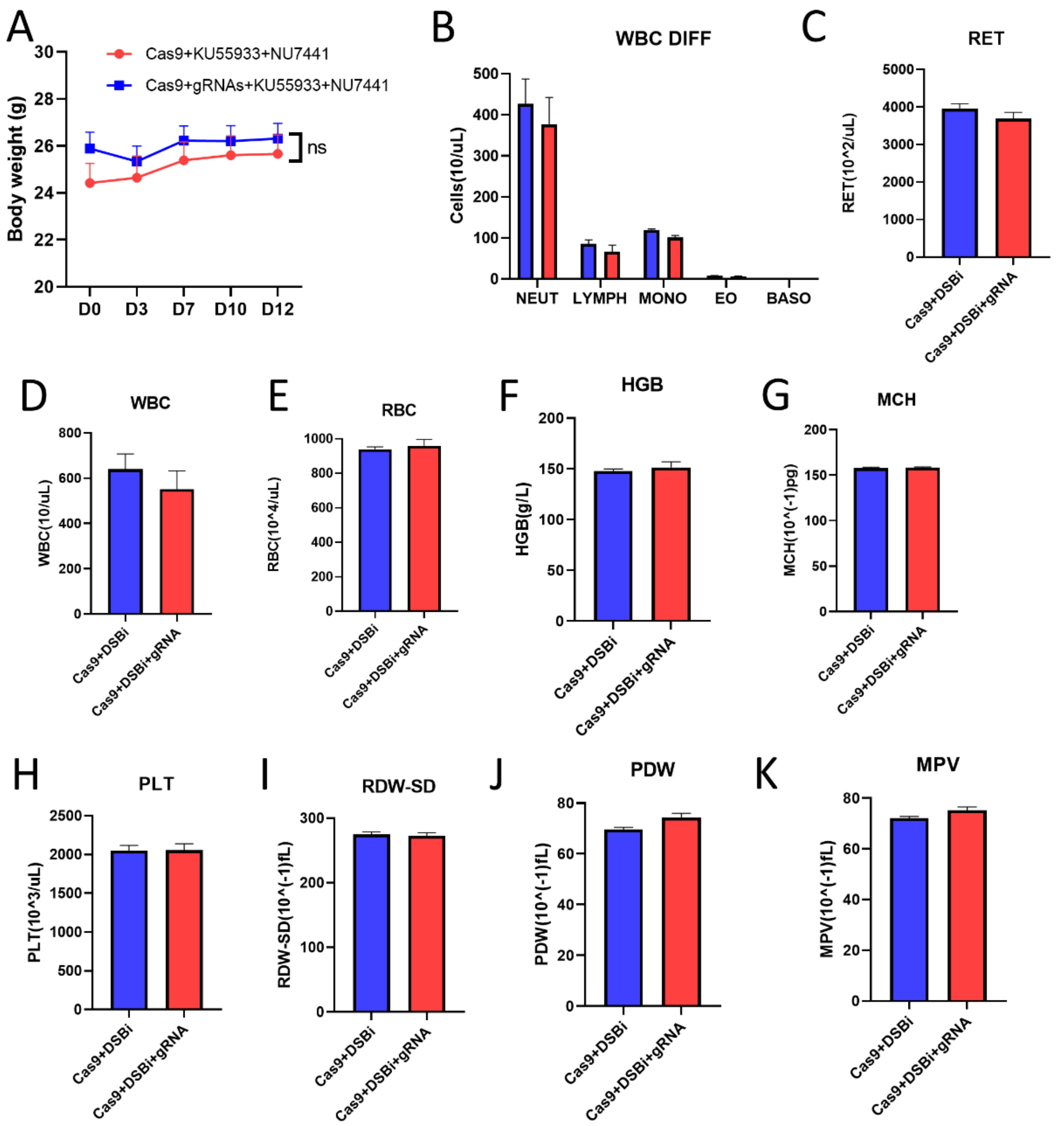

Figure S10. The "i-CRISPR " strategy could not significantly affect the weight, blood routine and other biochemical indicators of PCa PDX mice.

A. The body weight of PDX bearing mice were recorded every three days after the injection of gRNA and DSB inhibitor.

B-K. The blood routine test and biochemical parameters were measured on D12 after the application of CRISPR cutting strategy. No significant difference was observed in the two groups.

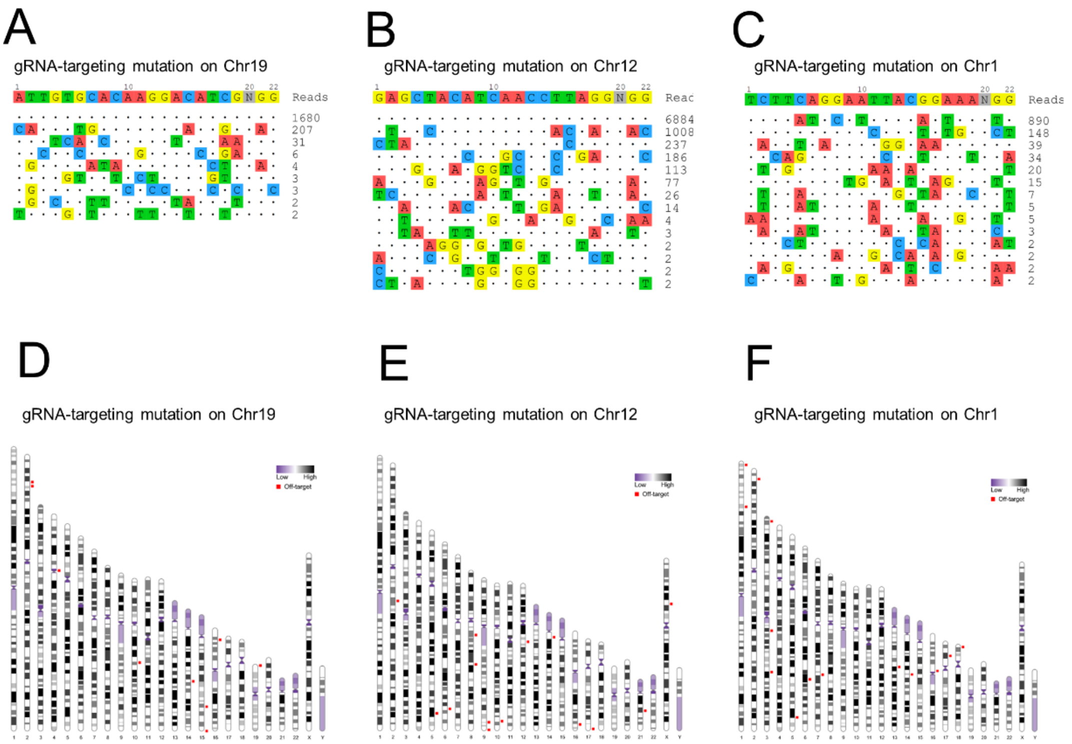

Figure S11. Sequences of off-target sites identified by GUIDE-seq for the mixture of Cas9 and 3 gRNAs targeting mutated sites and treated DU145 cells.

A-C. On-target and off-target efficiencies with gRNA targeting the mutation located on Chr19 (A), Chr12 (B) and Chr1 (C) evaluated using GUIDE-seq.

D-F. Off-targets distributions on all the chromosomes with gRNA targeting the mutation located on Chr19 (D), Chr12 (E) and Chr1 (F) evaluated using GUIDE-seq.

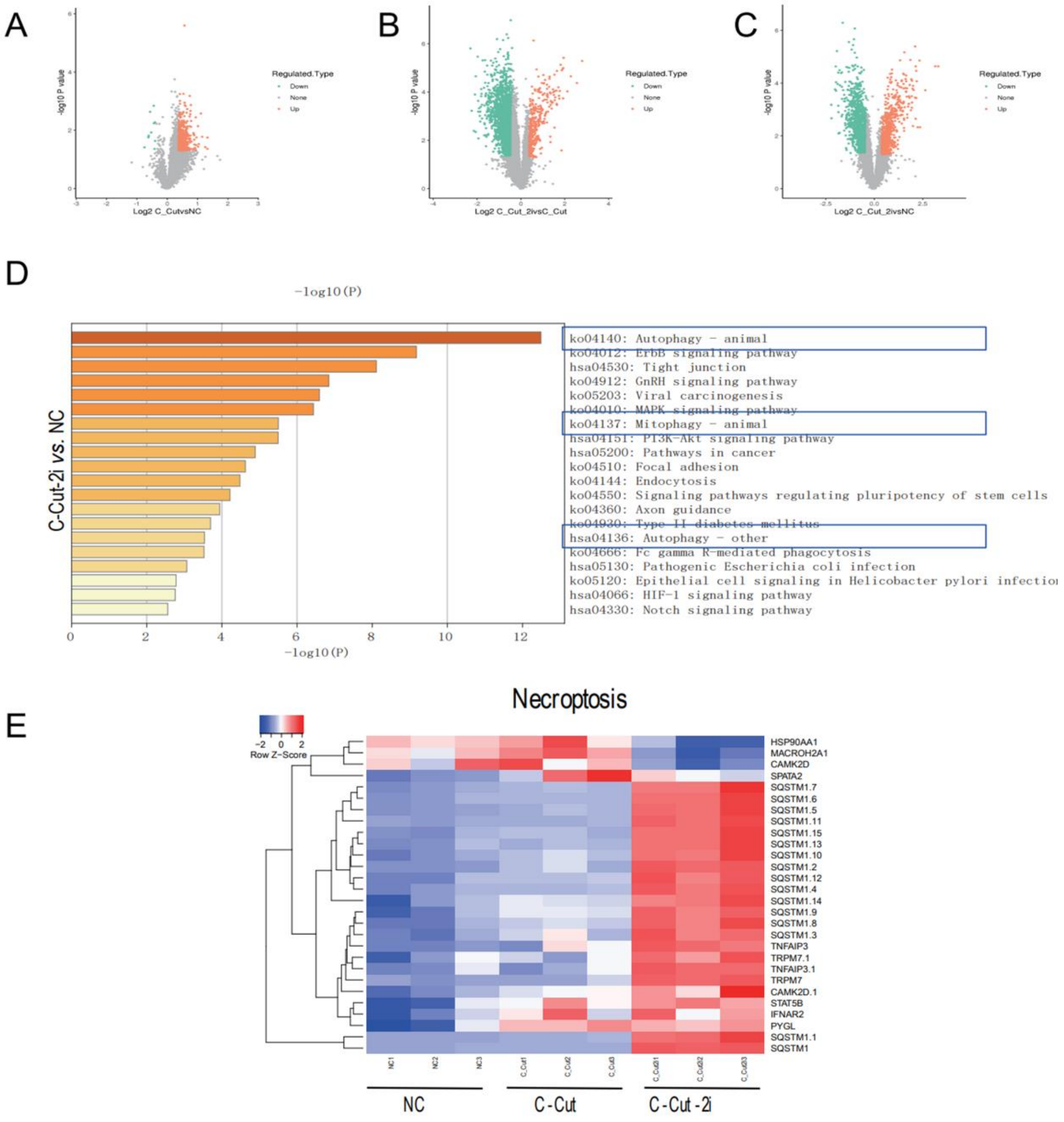

Figure S12. Quantitative phos-phoproteomics analysis elucidate the molecular mechanism of the "i-CRISPR " strategy in HepG2 cells

A-C. Volcano plot shows differentially phosphorylated proteins between the C-Cut and NC groups (A), C-Cut-2i and NC groups (B), and C-Cut-2i and C-Cut groups (C) after quantitative phosphoproteomics analysis using tandem mass spectrometry. NC: negative control, only treated with Cas9; C-Cut: CRISPR-Cut, treated with Cas9 and the 8 gRNAs in the T8 set; C-Cut-2i，CRISPR-Cut treated with the T8 set and 2i (NU7441 and KU55933).

D. Enrichment of GO terms for the proteins with upregulated phosphorylation between the C-Cut-2i and NC groups.

E. Heatmap shows the selected differentially phosphorylated proteins related to necroptosis among the 3 groups.

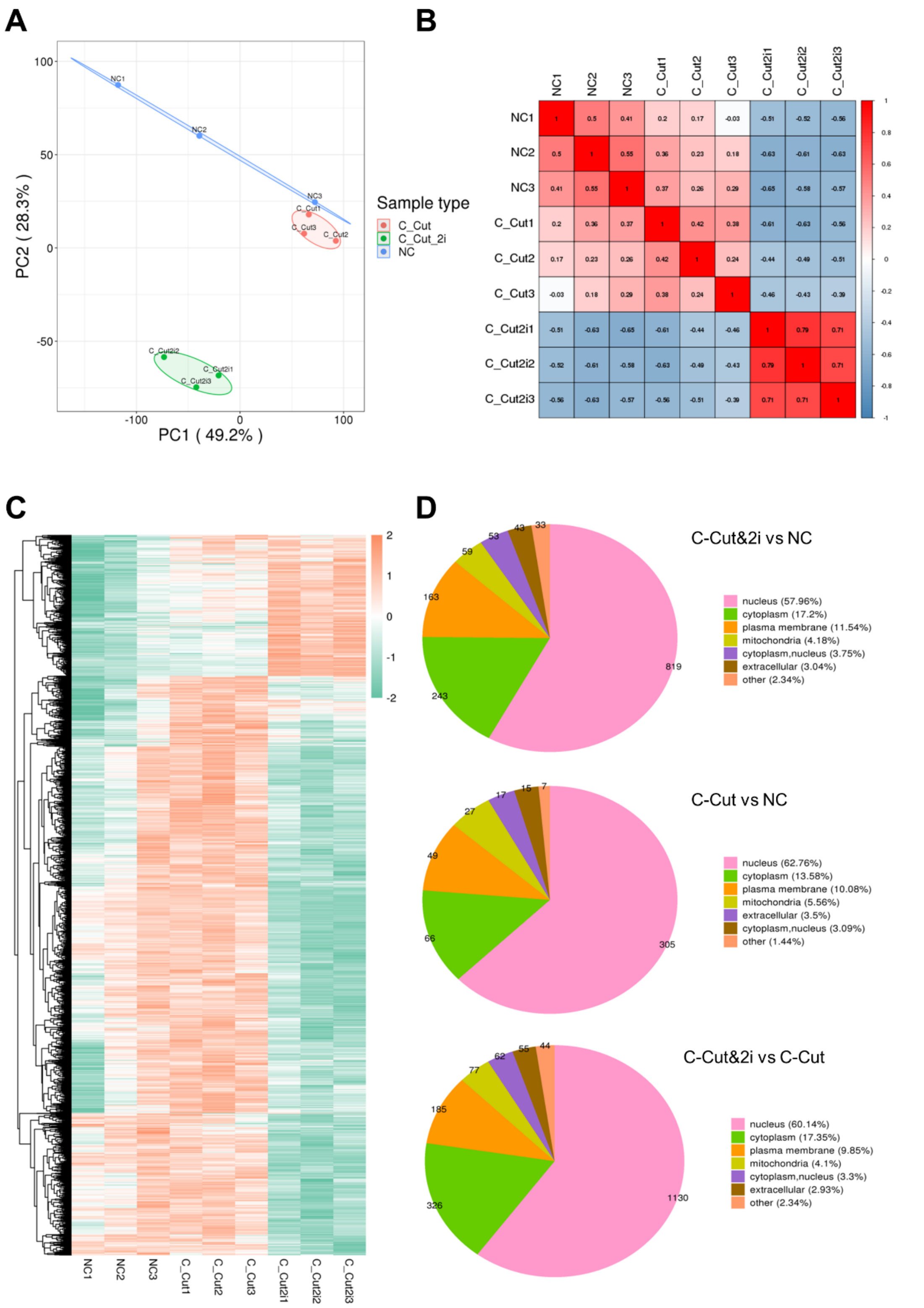

Figure S13 Differentially phosphorylated proteins analysis after the "i-CRISPR" strategy treatment.

A. The principal component analysis (PCA) result of the 9 samples (each group has 3 samples) after quantitative phos-phoproteomics analysis using the Tandem Mass. NC: negative control, only treated with Cas9; C-Cut: CRISPR-

Cut, treated with Cas9 and 8gRNAs in the T8 set; C-Cut&2i，CRISPR-Cut with T8 set and 2 inhibitors (NU7441 and KU55933).

B. Pearson correlation coefficients between all samples are shown in Heatmap.

C. Heat map shows the landscape of differentially phosphorylated proteins among the 3 groups.

D. Pie chart shows the distribution of differentially phosphorylated proteins in cell components based on GO analysis.

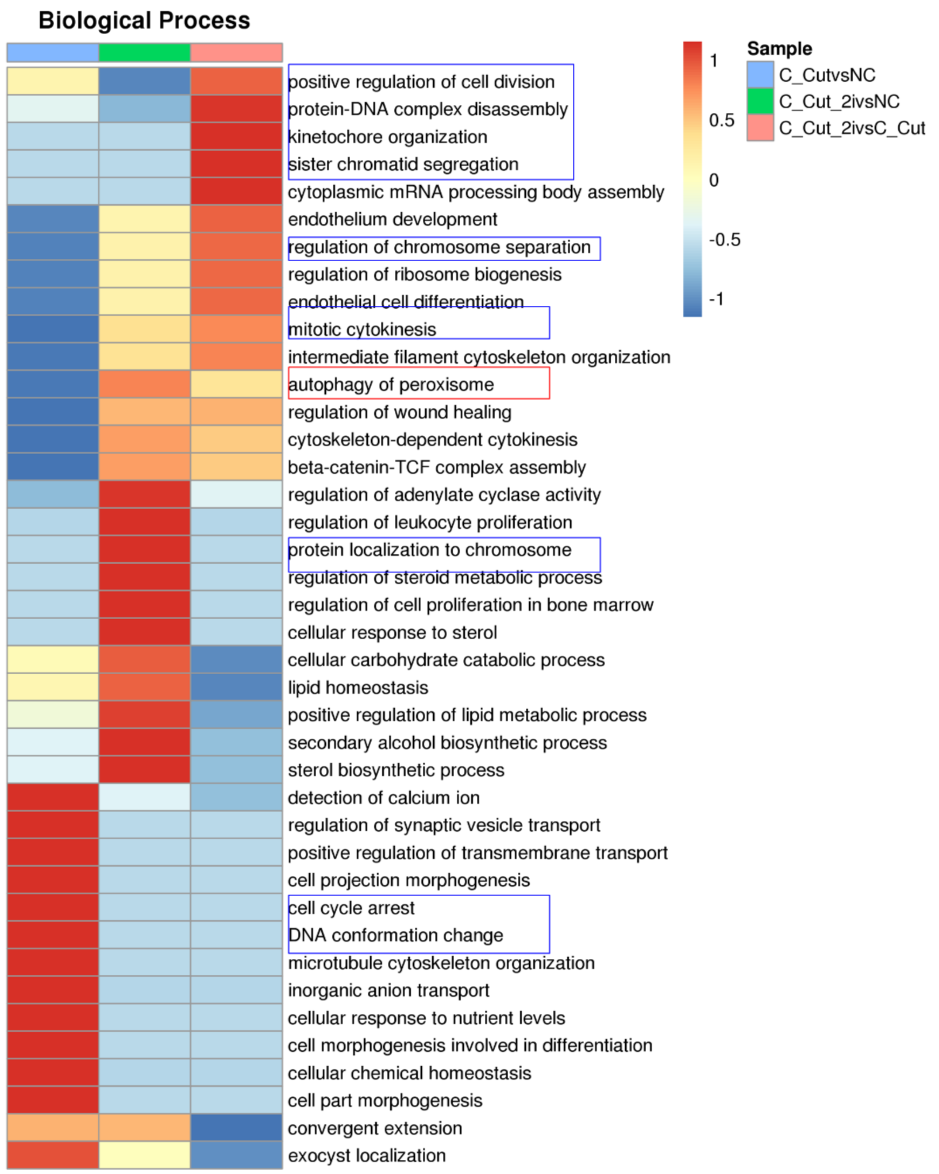

Figure S14 Enrichment of GO terms for differentially phosphorylated proteins among the 3 groups.

Enrichment of GO terms for differentially phosphorylated proteins among the 3 groups after quantitative phos-phoproteomics analysis using the Tandem Mass. NC: negative control, only treated with Cas9; C-Cut: CRISPR-Cut, treated with Cas9 and 8gRNAs in the T8 set; C-Cut&2i，CRISPR-Cut with T8 set and 2 inhibitors (NU7441 and KU55933).

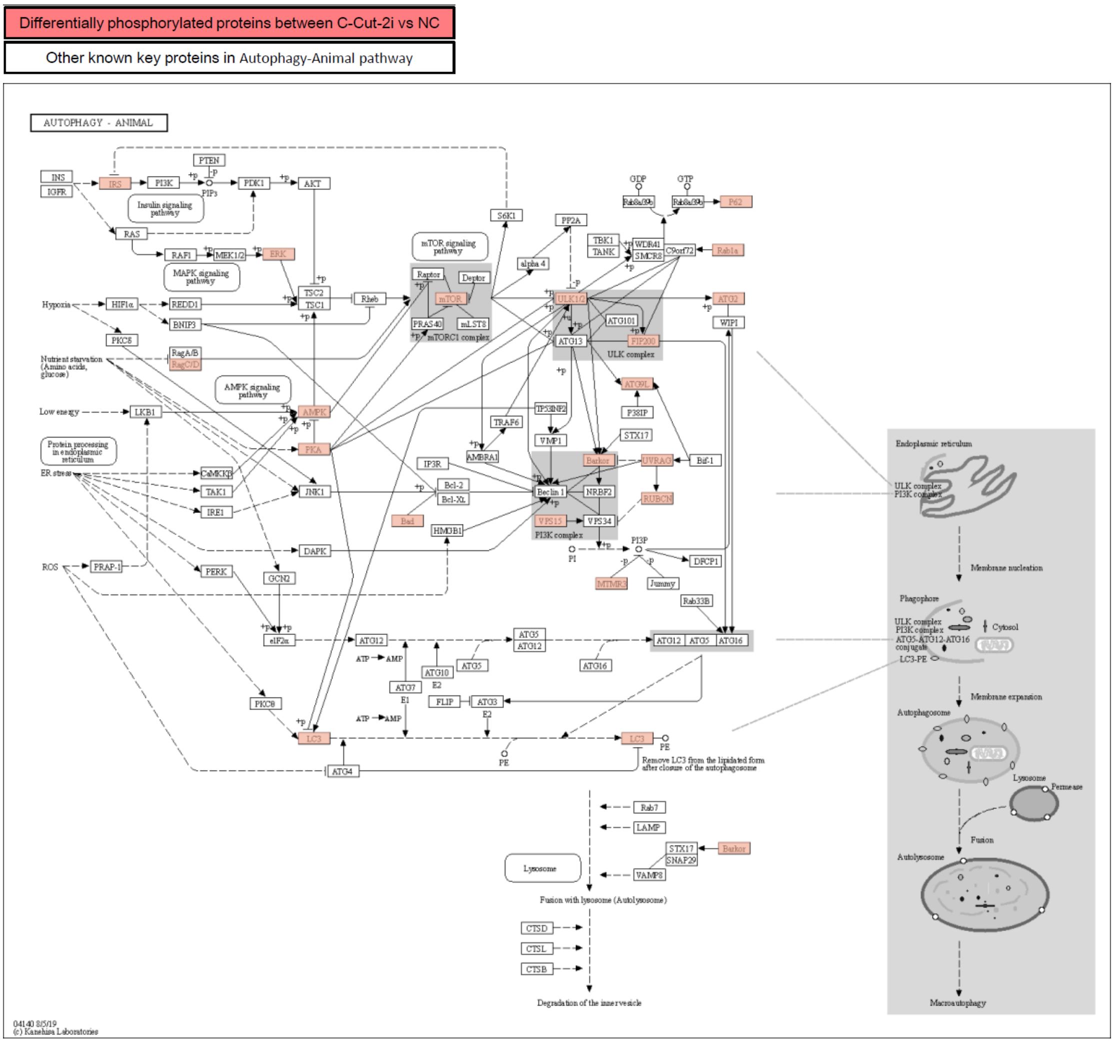

Figure S15. Activation of autophagy mayplay critical role in the molecular mechanism for the increased cell death of our strategy-part 1

KEGG pathway of the enriched term “Autophagy-Animal” in GO analysis， and differentially phosphorylated proteins are highlighted in the C-Cut-2i vs NC. Differentially phosphorylated proteins were analyzed using the Tandem Mass. NC: negative control, only treated with Cas9; C-Cut-2i， CRISPR-Cut with T8 set and 2 inhibitors (NU7441 and KU55933).

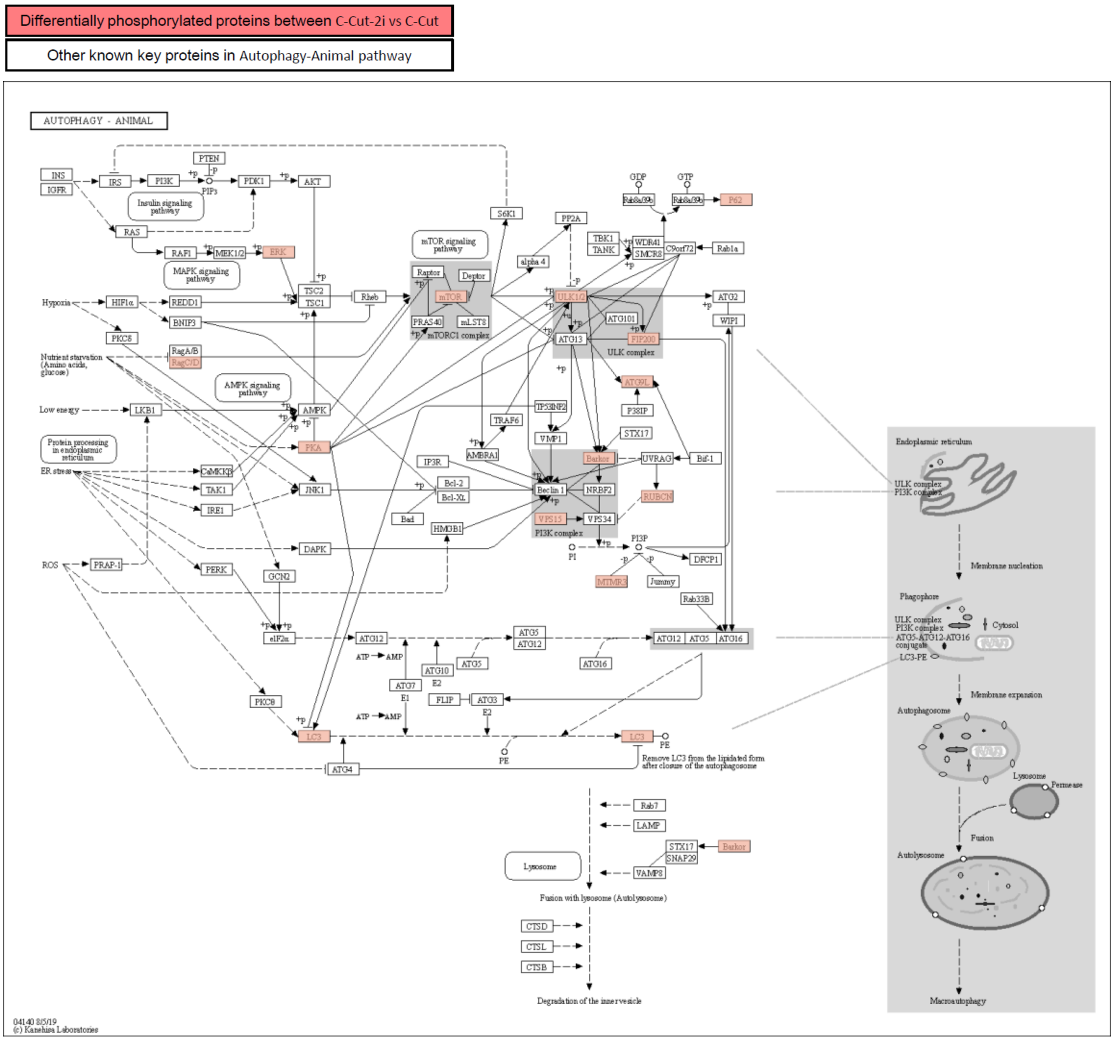

Figure S16. Activation of autophagy mayplay critical role in the molecular mechanism for the increased cell death of our strategy-part 2

KEGG pathway of the enriched term “Autophagy-Animal” in GO analysis， and differentially phosphorylated proteins are highlighted in the C-Cut-2i vs C-Cut. Differentially phosphorylated proteins were analyzed using the Tandem Mass. C-Cut: CRISPR-Cut, treated with Cas9 and 8gRNAs in the T8 set; C-Cut-2i，CRISPR-Cut with T8 set and 2i (NU7441 and KU55933).

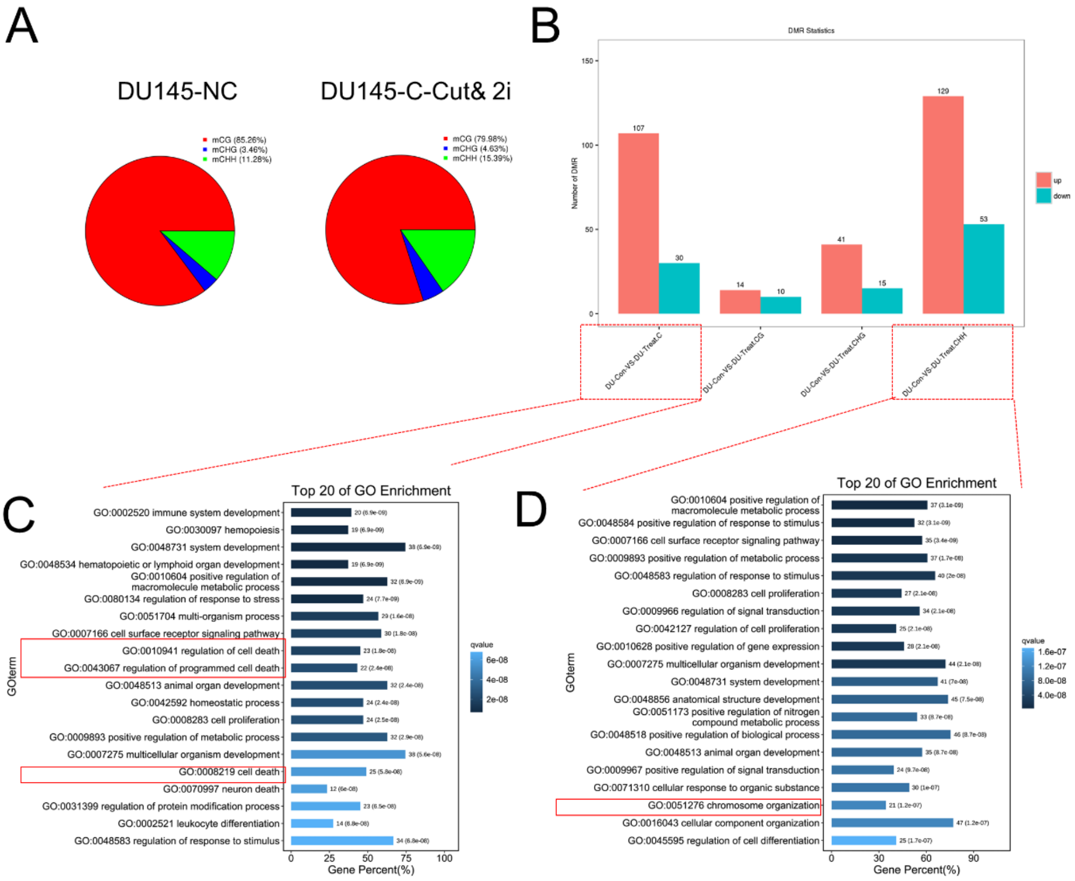

Figure S17 DNA methylation analysis in control and treated DU145 cells.

A. Pei chart shown the change of the composition ratio of methylation levels of CG, CHG, and CHH contexts in the two groups of DU145. NC: negative control, only treated with Cas9; C-Cut&2i， CRISPR-Cut with 7gRNAs and 2 inhibitors (NU7441 and KU55933).

B. differentially methylated regions (DMRs) numbers of the two comparison groups. DU-con:Du145 cells only treated with Cas9; DU-treat: Du145 cells treated with Cas9 and 7gRNAs（CRISPR-Cut and 2 inhibitors (NU7441 and KU55933);

C. Enrichment of GO terms for the genes located on the differentially methylated regions (DMRs) of C between the two comparison groups.

D. Enrichment of GO terms for the genes located on the differentially methylated regions (DMRs) of CHH between the two comparison groups.

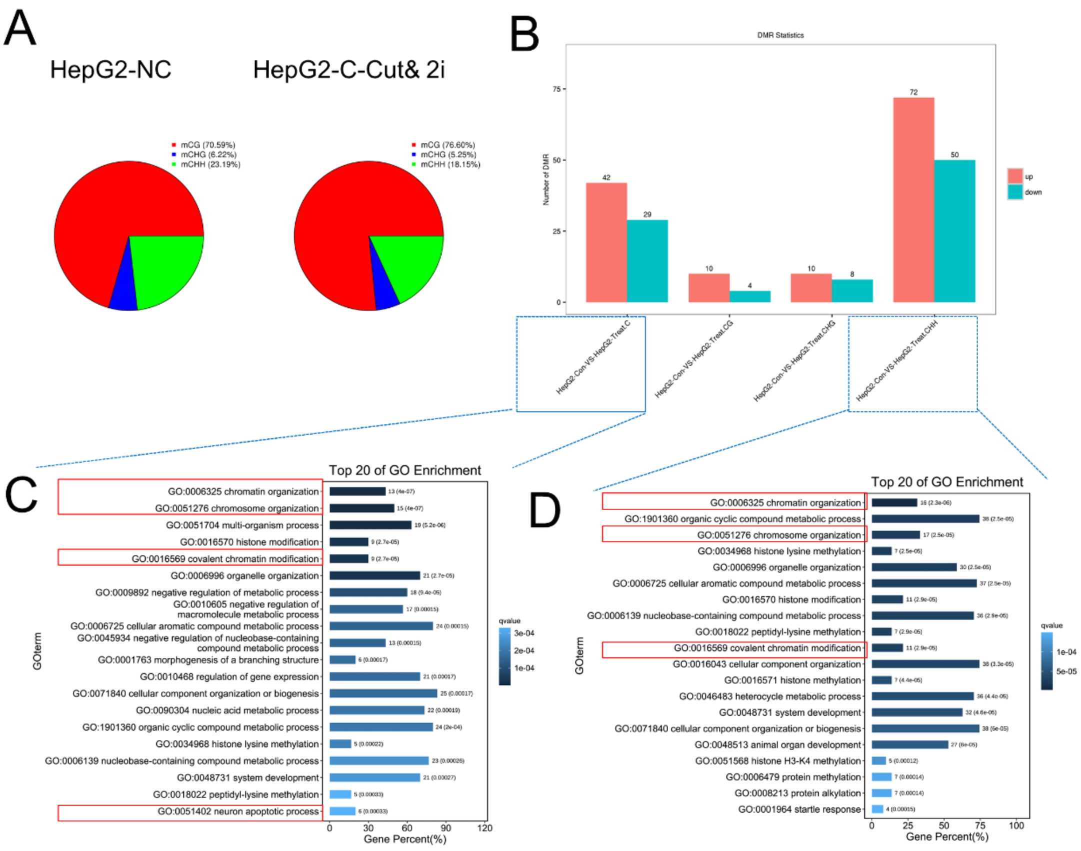

Figure S18 DNA methylation analysis in control and treated HepG2 cells.
A. Pei chart shown the change of the composition ratio of methylation levels of CG, CHG, and CHH contexts in the two groups of HepG2. NC: negative control, only treated with Cas9; C-Cut&2i，CRISPR-Cut with 8gRNAs and 2 inhibitors (NU7441 and KU55933).
B. differentially methylated regions (DMRs) numbers of the two comparison groups. HepG2-con: HepG2 cells only treated with Cas9; HepG2-treat: HepG2 cells treated with Cas9 and 8gRNAs（CRISPR-Cut and 2 inhibitors (NU7441 and KU55933);
C. Enrichment of GO terms for the genes located on the differentially methylated regions (DMRs) of C between the two comparison groups.
D. Enrichment of GO terms for the genes located on the differentially methylated regions (DMRs) of CHH between the two comparison groups.

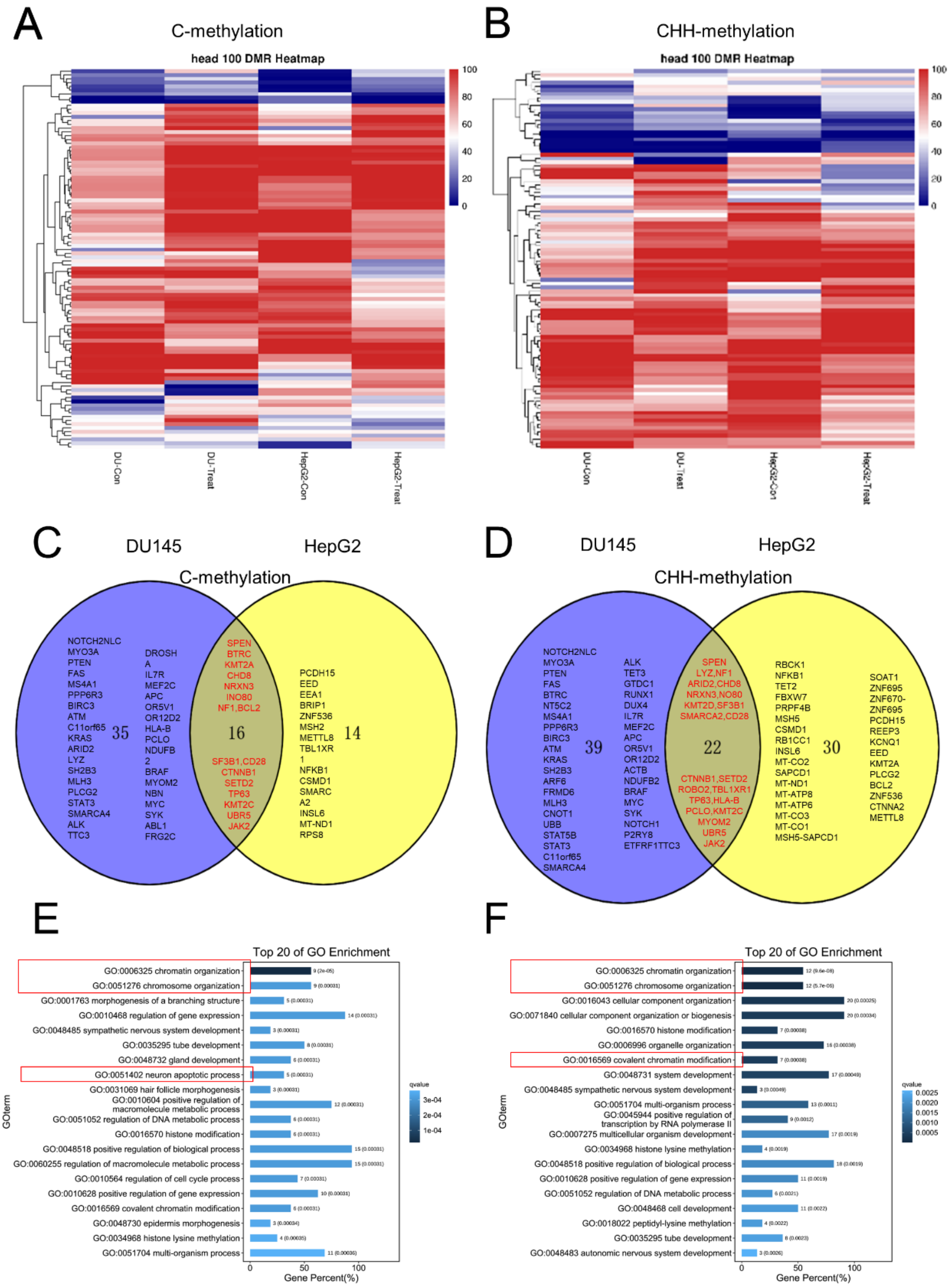

Figure S19 Joint analysis of the DNA methylation data in DU145 and HepG2 cells.

A. Heat map shown the of the regional methylation rate of the top 100 DMRs of C. DU-con: DU145 cells only treated with Cas9; DU-treat: DU145 cells treated with Cas9 and 7gRNAs CRISPR-Cut and 2 inhibitors (NU7441 and

KU55933); HepG2-con: HepG2 cells only treated with Cas9; HepG2-treat: HepG2 cells treated with Cas9 and 8gRNAs（CRISPR-Cut and 2 inhibitors (NU7441 and KU55933);

B. Heat map shown the of the regional methylation rate of the top 100 DMRs of CHH.

C. Venn diagram shows the common and specific DMRs of C located genes in DU145 and HepG2. Genes in the intersection are genes have DMRs of C in both DU145 and HepG2.

D. Venn diagram shows the common and specific DMRs of CHH located genes in DU145 and HepG2. Genes in the intersection are genes have DMRs of CHH in both DU145 and HepG2.

E. Enrichment of GO terms for the genes (in the intersection of C) that have DMRs of C in both DU145 and HepG2.

F. Enrichment of GO terms for the genes (in the intersection of D) that have DMRs of CHH in both DU145 and HepG2.

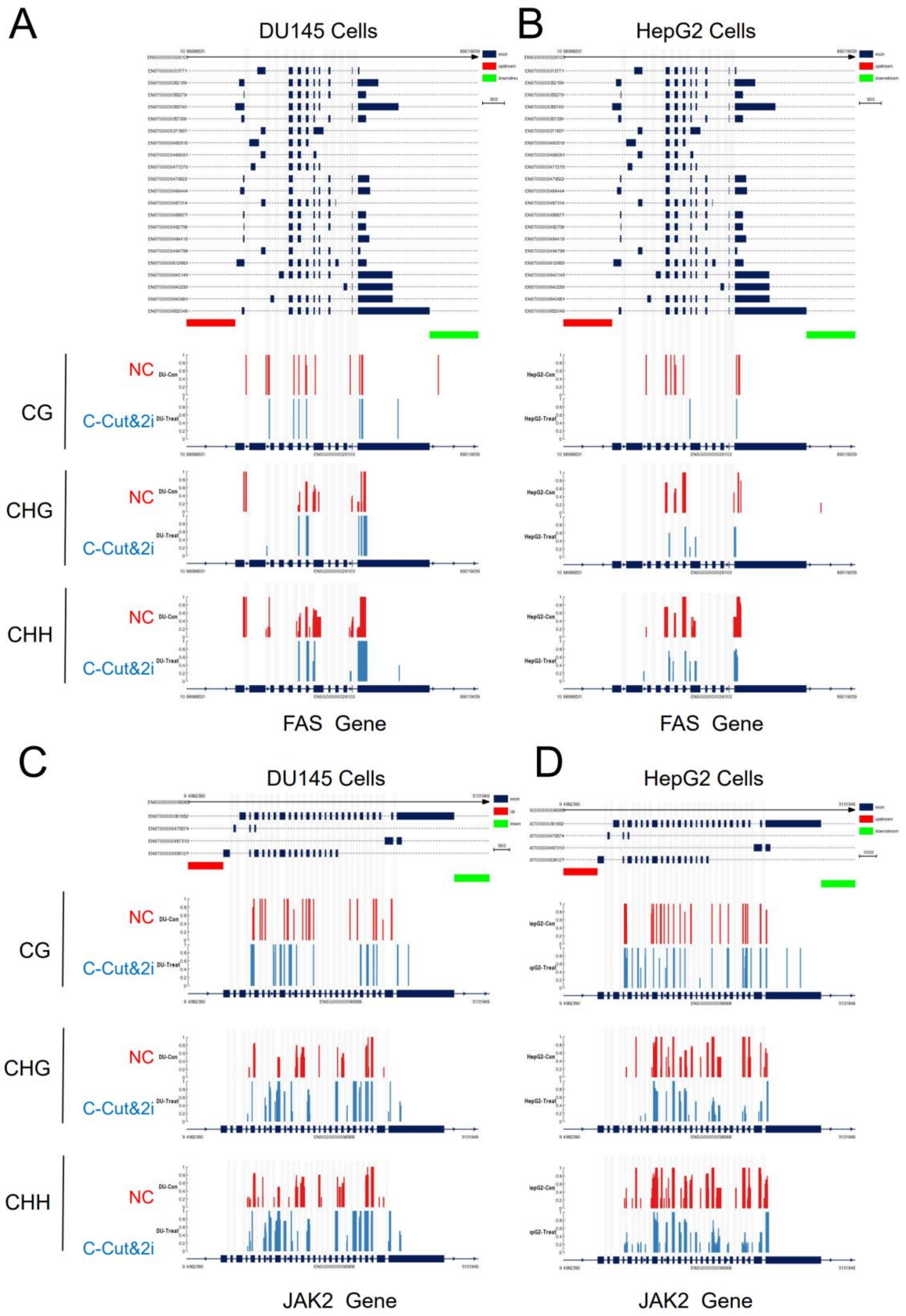

Figure S20 Gene body region methylation of representative DMR located genes in DU145 and HepG2 cells.

A. IGV software depicts the methylation of FAS gene body region in two groups of DU145 cells.NC: negative control, only treated with Cas9; C-Cut&2i， CRISPR-Cut with 7gRNAs and 2 inhibitors (NU7441 and KU55933).
B. IGV software depicts the methylation of FAS gene body region in two groups of HepG2. NC: negative control, only treated with Cas9; C-Cut&2i，CRISPR-Cut with 8gRNAs and 2 inhibitors (NU7441 and KU55933).
C. IGV software depicts the methylation of JAK2 gene body region in control DU145(DU-Con) and gRNA and 7 gRNAs-Cas9 treated DU145(DU-Treat).
D. IGV software depicts the methylation of JAK2 gene body region in control HepG2(HepG2-Con) and gRNA and 8 gRNAs-Cas9 treated HepG2(HepG2-Treat).

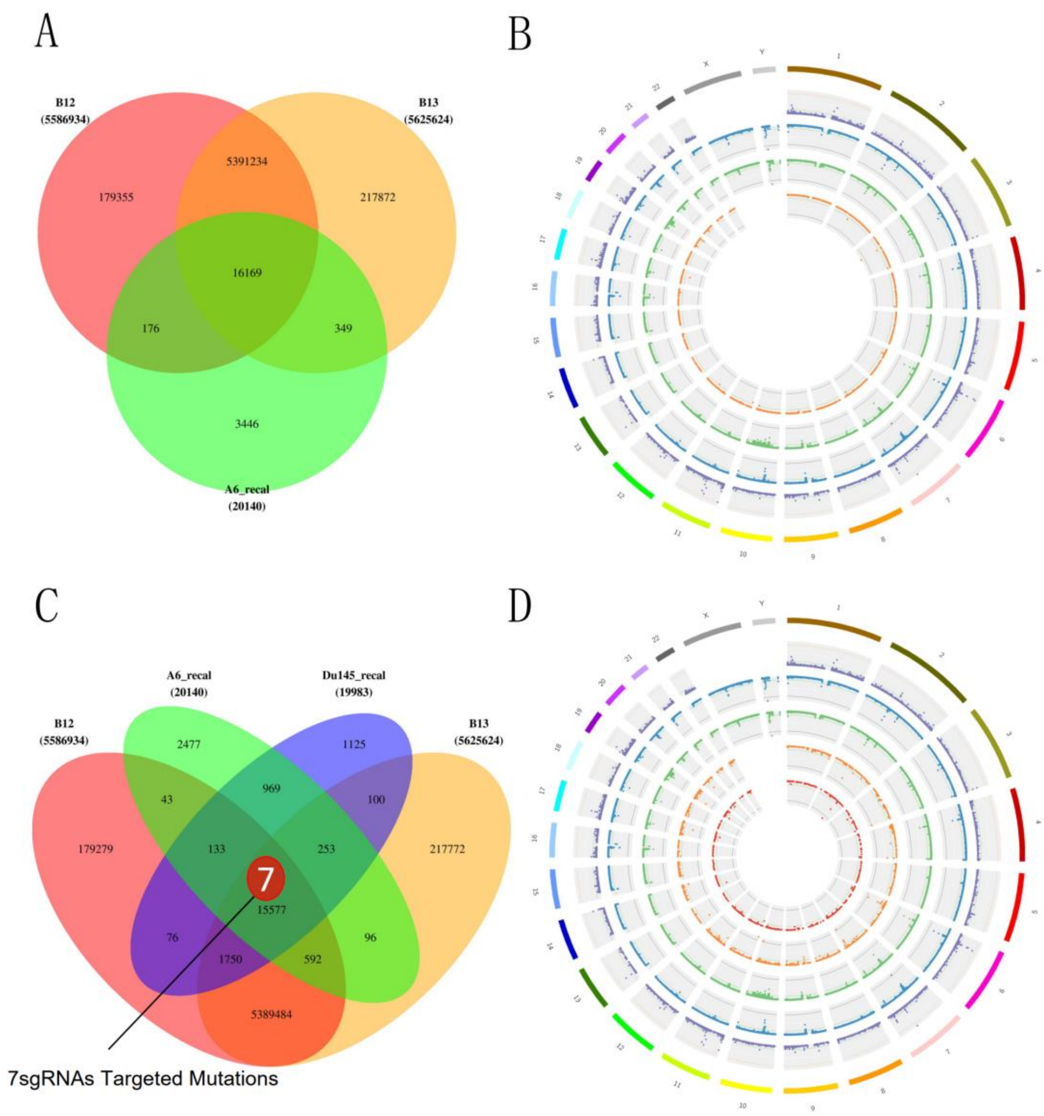

Figure S21 Whole-genome sequencing results of DU145 and the 3 DU145 derived single cells (A6 ，B12，B13) cultured in our laboratory

A. Venn diagram shows the common and specific mutations in the 3 DU145 derived single cells (A6 ，B12，B13) discovered by whole genome sequencing (WGS). A6 is a single-cell clone derived from DU145 that has been cultured alone for approximately 60 passages in our laboratory. From outside to inside：common and specific mutations in B12, B13 and A6. A6 is a single-cell clone derived from DU145 that has been cultured alone for approximately 60 passages in our laboratory. B12 and B13 are two single-cell clones derived from DU145 that has been cultured alone for approximately 80 passages in our laboratory.

B. Circos plot showing the distribution of the common and specific mutations in B12, B13 and A6. From outside to inside：common and specific mutations in B12, B13 and A6.

C. Venn diagram shows the common and specific mutations in DU145 and the 3 DU145 derived single cells (A6 ，B12，B13) discovered by WGS. From outside to inside：common and specific mutations in B12, B13 and A6.

D. Circos plot showing the distribution of the common and specific mutations in DU145，A6，B12, and B13. From outside to inside：common and specific mutations in B12, B13, A6 and DU145.

2. Supplementary materials and methods

Materials and methods

DNA sequencing and CRISPR-Cas9 gene editing

The WGS (whole genome sequencing) and was conducted by OE Biotech Co., Ltd. (Shanghai, China). TruSeq Nano DNA LT Sample Prepararion Kit (Illumina, San Diego, CA, USA) was used for DNA libraries construction. Illumina sequencing platform HiSeq X Ten platform (Illumina Inc., San Diego, CA, USA) was used for WGS of the libraries. Data analysis was also conducted by OE Biotech Co., Ltd. (Shanghai, China).

In our criteria, the sgRNAs were selected manually based on the following criteria: 1. High specificity in tumor cells; 2. Each site including 3 continuous base mutations. 3. For cell lines, the sites were both identified in sequencing and public database, which could be present in early passages of cells and shared by most labs in the world. 4. The length of indel bases were also considered for cutting purpose. For the design of each gRNA targeting the selected specific mutated DNA site in tumor cells, we first uploaded the mutated sequence to the gRNA design website CRISPick (https://portals.broadinstitute.org/gppx/crispick/public).Then, we used NCBI BLAST to exclude the gRNA sequences that could target any other human normal genomic DNA loci. Then, the selected gRNA sequence targeting mutation in HepG2, DU145 and Hep3B cells was synthesized and cloned into adenovirus expression vector h9350 (padeno-u6-spgrnav2.0-cmv-sfgfp-p2a-3flag-spcas9，Obio Technology, Shanghai) after U6 promotor. And the plasmid expressing both gRNA and Cas9 was then packaged into adenovirus by Obio Technology, Shanghai. The selected gRNA sequence targeting mutation in DU145 was synthesized and cloned into lenti-virous expression vector H6805, pCLenti-U6-spgRNA v2.0-CMV-EGFP-F2A-BSR-WPRE (Obio Technology, Shanghai) after U6 promotor. And the plasmid expressing gRNA was then packaged into lenti-virus (Obio Technology, Shanghai). The usage of the virus was in accordance with the company's instructions and adenovirus safe use

principles. All the target sites as well as gRNA sequences in HepG2 cells, Hep3B cell, DU145 cells, organoids and PDX model were listed in the supplementary tables.

Cells and treatments

Human liver carcinoma HepG2 cells, Hep3B cells, Huh-7 cells and prostate cancer DU145 cells were purchased from ATCC (USA). Human embryonic kidney cells were purchased from the ATCC and maintained in our lab. Cells were maintained in DMEM or RPMI1640 medium supplemented with 10% fetal bovine serum at 37℃ in a 5% CO_2 humidified incubator. For delivering gRNA and Cas9 into HepG2 cells and Huh-7 cells, cells were infected with adenovirus carrying Cas9 and the designed gRNAs targeting the HepG2 mutated sequences in the following group: 4 Targets set 1 (T4-set 1, mixture of 4 Cas9-gRNAs adenovirus targeting 4 HepG2 mutated sites); 4 Targets set 2 (T4-set 2, mixture of another 4 Cas9-gRNA adenovirus targeting another 4 HepG2 mutated sites); 8 Targets set (T8-set,mixture of all the 8 Cas9-gRNA adenovirus targeting all the 8 HepG2 mutated sites)and NC control group(NC, negative control gRNA sequence).For delivering gRNA and Cas9 into DU145 cells, cells were infected with lentivirus in the following groups: 3gRNAs(mixture of 3 gRNA lentivirus targeting 3 DU145 mutated sites),4gRNAs(mixture of 4 gRNA lentivirus targeting 4 DU145 mutated sites), 7gRNAs(mixture of all the 7 gRNA lentivirus targeting the 7 selected DU145 mutated sites) and NC groups. And another Cas9-expressing lentivirus was added into all 4 groups. For delivering gRNA and Cas9 into Hep3B cells, cells were infected with lentivirus carrying Cas9 and the designed gRNAs targeting 4 Hep3B mutated sequences. To verify the cutting efficacy of the gRNAs, PCR products of specific DNA sites from HepG2 cells introduced with 8gRNAs, DU145 cells with the 7gRNA, and Hep3B cells with the 4gRNAs and Cas9 were subjected to Sanger sequencing with (Applied Biosystems, ABI3730).

For inhibition of DNA damage repair in HepG2, adenovirus infected cells were

treated with ATM inhibitor KU55933, DNA-PKcs inhibitor NU7441 and the combination of “KU55933+NU7441”. For inhibition of DNA damage repair in DU145, lentivirus infected cells were treated with PI3KK inhibitor Wortmannin. To test the resistance resulted from the first-round treatment, DU145 cells treated with first set of sgRNAs were further treated with the same group of sgRNA or a different set of sgRNA for a second round. At indicated time points after transfection, cells were treated and used for next experiments.

Organoids and treatment

As previously described, patient derived organoids (PDO) of hepatobiliary tumor were established before and were applied to test the anti-tumor efficacy of our strategy [1]. Briefly, the organoids were kindly provided form our collaborators Prof. Lei Chen from Eastern Hepatobiliary Surgery Hospital in our university. DNA sequencing were performed in the organoids (HCC227) and targeting sgRNAs were designed. Cells of organoids were plated into 384 well plates and after 3 days culture, organoids were infected with lentivirus expressing only Cas9 protein or Cas9 plus sgRNAs. Then the images were taken with a light microscope every day up to 7 days. Alternatively, CCK-8 assay was also used to measure the viability of organoids. Data from three independent experiments were statistically analyzed.

Animal experiments and patient derived xenograft (PDX) models

The xenograft experiments were performed using nude mice. As we reported previous, prostate cancer cells DU145 cells were subcutaneously injected into the 4-week-old male nude mice [2]. Each nude mouse received 3×10^6 cells in 100 μl of serum-free medium blended with an equal volume of BD Matrigel Matrix (BD Biosciences). After 3 weeks, the mixture of 4 gRNAs lentivirus targeting 4 DU145 mutated sites (treated group) or NC lentivirus were injected intratumorally with Wortmannin and Cas9-expressing lentivirus. The injections were given every three days for a total of four times. Mice were sacrificed at6

weeks. All procedures were obeying to the Animal Care and Use Committee of the Naval Medical University, Shanghai, China.

For PDX model, tumor tissues from patients with prostate cancer were resected surgically and implanted into mice with the technique assistance from Shanghai Lidi Co. Ltd as previously described [3]. Briefly, the tumor tissues were cut into size of 1 to 3 mm and implanted subcutaneously into the flank region of male NOG mice. the engrafted tumor tissues were passaged and implanted into different male NOG mice. At the same time, DNA sequencing were also performed in isolated tumor tissues, based on which the sgRNAs were designed and synthesized. Once the PDX model grew to 50 to 80 mm^3, lentivirus carrying sgRNAs and Cas9 were intratumorally injected, together with the DNA damage repair inhibitor (KU55933 and NU7441). The lentivirus combinations were injected every two days for three times. Then the tumor volumes were monitored up to 12 days after CRISPR cutting, and the blood routine examination as well as biochemical analysis were performed. At the end point of observation, tumors were resected and tumor weight were measured. the data were expressed as mean SEM, and statistical analysis were further performed (n=6).

Immunofluorescence staining and analysis

Immunofluorescence staining was used to detect γH2AX foci, the cleaved Caspase 3 and Caspase 3 by using methods described in our previous studies[4, 5]. Briefly, cells were seeded on 22X22mm^2 cover glasses. After different treatments, cells were fixed in 4% paraformaldehyde for 20 min at room temperature, followed by treatment with 0.5% Triton X-100 for permeabilization. Then the slides were blocked with goat serum and incubated with primary antibodies as follows: γH2AX S139 (CST, 1:1000), c-Caspase 3 (Abcam, 1:500), Caspase 3 (CST, 1:200). Then the fluorescence images were obtained with a Zesis LSM-880 confocal microscope in the State Key Laboratory of Genetic Engineering in Fudan University. Image pro plus (Media

Cybernetics) were used to count γH2AX foci and analyze the fluorescence density.

Cell apoptosis

At 48h and 72h after CRISPR-virus infected and treatments with DNA damage repair inhibitors, cells were collected and stained with Annexin V-FITC and PI apoptosis kit according to the manufacture's instructions (Yeasen, Shanghai, China). After Annexin V-FITC and PI double staining, cells were analyzed with a cytoflex flow cytometry (Beckman cytoflex, USA).

Cell viability

At 0, 24, 48h and 72h after CRISPR-virus infected and treatments with DNA damage repair inhibitors, cells were stained with CCK-8 solution and cell viability was determined with a BioTech reader at the wavelength of OD450. For organoids experiments, CCK-8 solution was added into the wells with organoid and incubated with 4-8 h, after then the wavelength of OD450 were detected with a plate reader.

Phos proteomics

The HepG2 cells were divided into three groups: NC, C-Cut and C-Cut-2i, with three samples in each group. The NC (negative control) group was only treated with Cas9; the C-Cut（CRISPR-Cut）group was treated with Cas9 and 8gRNAs in the T8 set; and the C-Cut-2i（CRISPR-Cut& 2i）group was treated with Cas9 and 8gRNAs in the T8 set and 2 DNA damage repair inhibitors (NU7441 and KU55933). After 72 hours of treatment, the cells were harvested and prepared for phos-phoproteome analysis.

Briefly, an integrated approach involving TMT kit (Thermo Fisher) labeling, high performance liquid chromatography (HPLC) fractionation, immobilized metal affinity chromatography (IMAC) affinity enrichment, and LC-MS/MS using a Q Exactive™ HF-X Hybrid Quadrupole-Orbitrap mass spectrometer coupled with

EASY-nLC 1000 liquid chromatography pump (Thermo Fisher Scientific) was employed to quantify the dynamic changes in the whole phos-phoproteome of the three groups of cells. Both methods were performed with the support of PTM-Biolabs Co. Ltd. (310018; HangZhou, Zhejiang, China). Metascape webtool (www.metascape.org) was used to conduct Gene ontology (GO) biological process and Kyoto Encyclopedia of Genes and Genomes (KEGG) pathway analysis, allowing us to visualize the functional patterns of DEGs and conduct statistical analysis.

Whole-Genome Bisulfite Sequencing

The HepG2 cells were divided into two groups: NC and C-Cut-2i. The NC (negative control) group was only treated with Cas9, and in the C-Cut-2i （CRISPR-Cut& 2i）group, mixture of the Cas9-gRNA adenovirus targeting the selected 8 HepG2 mutated sites and 2 DNA damage repair inhibitors (NU7441 and KU55933) were added into cultured HepG2 cells. Similarly, the DU145 cells were also divided into two groups: NC and C-Cut-2i. The NC (negative control) group was only treated with Cas9, and the C-Cut-2i（CRISPR-Cut& 2i）group was treated with Cas9 lentivirus and mixture lentivirus of the 7 gRNAs and 2 DNA damage repair inhibitors (NU7441 and KU55933). Three days after treatment，cells were harvested and genomic DNA was extracted using the QIAamp Mini DNA kit (Qiagen,) according to the instructions. The DNA concentration were detected by NanoDrop spectrophotometer and fragmented into 100-300bp by sonication (Covaris, USA) and purified before end repairing and a single “A” nucleotide adding onto the 3’ end of the blunt fragments. Then the DNA fragments were ligated to methylated sequencing adapters， and were bisulfite converted using Methylation-Gold kit. The converted DNA were amplified and sequenced using Illumina HiSeqTM 2500 and the Data were analysed by Gene Denovo Biotechnology Co. (Guangzhou, China).

Off-target detection by Guide-seq

DsODN (200pmol) was transfected into cultured DU145 cells(2×10^6) by Lipofectamine 3000 (Invitrogen), and then Cas9 and gRNA lentivirus targeting 3 DU145 mutated sites were added into the cells. Three days after treatment， cells were harvested and genomic DNA was extracted using the QIAamp Mini *DNA* kit (Qiagen) according to the instructions. Library construction, Guide sequencing and data analysis was performed by GeneRulor Company Bio-X Lab, Guangzhou 510006, Guangdong, China.

Statistical analysis

Data were expressed as the means ± standard error of mean (SEM). GraphPad Prism 8 software was applied for statistical analysis as previously described. Briefly, one was ANOVA was used for comparison among multiple groups. For comparison between two groups, Student's t test was used. $P<0.05$ was considered as statistically significant. All the experiments were performed at least 3 independent times.

1. Zhao, Y., et al., *Single-Cell Transcriptome Analysis Uncovers Intratumoral Heterogeneity and Underlying Mechanisms for Drug Resistance in Hepatobiliary Tumor Organoids.* Adv Sci (Weinh), 2021. **8**(11): p. e2003897.
2. Xiao, G., et al., *The Long Noncoding RNA TTTY15, Which Is Located on the Y Chromosome, Promotes Prostate Cancer Progression by Sponging let-7.* Eur Urol, 2019. **76**(3): p. 315-326.
3. Yang, G., et al., *Integrative Genomic Analysis of Gemcitabine Resistance in Pancreatic Cancer by Patient-derived Xenograft Models.* Clin Cancer Res, 2021. **27**(12): p. 3383-3396.
4. Lei, X., et al., *Nuclear Transglutaminase 2 interacts with topoisomerase II[illegible] to promote DNA damage repair in lung cancer cells.* Journal of experimental & clinical cancer research : CR, 2021. **40**(1): p. 224.
5. Liu, L., et al., *Long non-coding RNA ANRIL promotes homologous recombination-mediated DNA repair by maintaining ATR protein stability to enhance cancer resistance.* Molecular cancer, 2021. **20**(1): p. 94.
